# Supplementary material for: The reality of embedded drug purchasing practices: Understanding the sociocultural and economic aspects of the use of medicines in Bangladesh
Source: PLoS One. 2023 Jun 28;18(6):e0287009. doi: 10.1371/journal.pone.0287009 (PMC10306190; doi:10.1371/journal.pone.0287009)
Supplement: S1 File — (DOCX) [file pone.0287009.s001.docx]

**S1. Research tools In-depth Interview and Key Informant Interview Guidelines, Observation checklist**

**Guidelines for Interview**

**Study Title:** The Complexity of Drug Transaction in Pharmacy: An Ethnography of Drug Sellers-Clients interaction in Community Pharmacy in Bangladesh

**Principal Investigator’s Name:** Md. Shahgahan Miah

**IDI guidelines for patient, client**

**Socio-demographic information about participant:** Age, gender, marital status, number of family members, religion, education, profession, income (monthly).

**Content of demands**

1. For whom and for what kind of illness bring you to the pharmacy?
2. What purposes do you visit to this drug store (buy medicine, seeking care, information, consult for visit/appointment a doctor/hospital/clinic)?
3. For what kind of illness do you visit this pharmacy usually, please describe in details?
4. Do you visit only this pharmacy? If yes (explore the possible reasons e.g. distance, availability, cost, known, trust,)? How long have you been seeking care, medicines from this pharmacy? Please explore in details if visit to other pharmacies? Where/how frequent and why?
5. Did you think the other factors influences you to make visit to this seller? What are these? Whom did you consulted or suggested to visit?
6. In your opinion, before visited to the pharmacy did you feeling bodily discomfort? If yes, how do you explain about your (self/family member/relatives) unwell situation when you visited?
7. What kinds of medicines do you buy from the drug store? Do you buy drug with prescription, without prescription, self-medication?
8. Did you buy medicine/drugs according to the prescription/consultation? (Probe: less amount of medicine, what are the reasons: financial barrier/providers attitude/not available/costly)
9. Could you please tell me your experiences about the purchase of drugs? (Probe: waiting time, buy instant what and when you feel need?
10. Would you please tell me about your experiences of how the provider ask, listen to your health problems and requirements when you visited?
11. Please share the drug decision process (asked the exact drug name, showing old samples/prescriptions, provider recommend, negotiated)?

**Factors stimulate to visit**

1. Could you please share what are the sources of your health information in case of you and your family members’ illness? Whom one you trust more? Why?
2. From your experiences please tell me what factors (e.g. belief, trust) influence you to visit pharmacy for seeking healthcare, drug purchase? Please describe elaborately (how and why/why not)?
3. In your opinion, what are the important elements/aspects that affect your drug choices in and around your preferred pharmacy/drug store?
4. Could you please describe your expectations (e.g. quality services, drug preference, low price) when you come to the pharmacy? How do you deal with your expectation and why?
5. Do you think there have any negotiation option to choice the drugs, dose, price? If yes, how you decide for drugs selection? If not, how providers practices their power role in drug decisions?
6. Could you please share your experiences of getting services from the drug store (Explore positive side: manner/well-behaved, reduced price, knowledgeable, available etc.) (Explore negative side: unexpected behavior, unskilled, unavailable drugs/not open, costing etc.)?

**Evaluate the implications**

1. How would you describe your interactions with the sales attendant/seller/provider/owner cum seller was?
2. Could you please share how this interaction for drugs affected your unwell health?
3. Do you read or ask about the drug’s side effects or reaction, expire date and price on the cover of the medicines? If yes/no, then why?
4. Would you please share, does the drug seller provide information (e.g. dose, potential side effect, course, price) to you appropriately/do you feel the needs to know this, yes/no please explore in details?
5. Do you know anything about the drug policy (e.g. prescription, over the counter (OTC) drug, self-medication) that followed by you and your provider as well?
6. From your own experiences, how could you want to be interacted for your treatment purpose visit when you come to drug store?
7. What are the barriers do you feel for drug transaction interaction? From your own understanding, how could overcome these barriers?
8. In conclusion, are there anything else haven’t discussed that you wish to say?

**IDI guidelines for Drug seller, sales attendant, pharmacist, little trained doctor cum seller, owner cum seller**

**Socio-demographic information about participant:** Age, gender, marital status, number of family members, religion, education, professional degree, training, profession, income (monthly), years of experiences, license status

**About Drug store/Pharmacy**

1. How long have you been in this profession?
2. Year of running the pharmacy?
3. Tell me your responsibilities in managing the drug store?
4. How do you become (choice, motivation, training) a drug provider/seller/owner cum seller?
5. Do you have any other business or income source parallel with this drug selling? If yes, what and why?
6. Do you listen to the patient-centric approach (PCA)? If yes, how? How do you apply your PCA knowledge during drug interaction with patients?
7. What are the process to get the permission for license certificate? What you need to go for that? What you need to follow? Does regulatory people monitor, what, how often?
8. From where you purchase your drugs (company, dealer or wholesaler, or retail shop)?

**About Drug dealing**

1. From your experiences, could you please tell me, for what kinds of drug demand, and health problems usually you deal with your clients?
2. Could you please tell me; what clients usually do (e.g. demand drugs by name, expectation, negotiation) when visited to the pharmacy?
3. How do you ask your clients about their health, drug related problems? Do you talk any greetings, social talk with your clients?
4. How your clients explain about their health, drug related problems?
5. Do you provide drugs without any written prescription? What is the process? (Probe: not use any prescription, mobile communication)
6. Could you please tell me from your experiences, do your clients have any expectation when they visited to you? If yes, how do you deal with their expectation?
7. Do you provide necessary information about drug uses (time, dose, course, side effects)? way of provide information: directly/mobile
8. Does your patient listen to you what you provide information about drug use? If yes/ no, why?
9. Do you have any health information sources (any physicians, medical promotion officer, newspaper, internet, mobile phone)? whom you trust more and why?
10. Please say something about your drug selection for your clients (previous experiences, suggestion from medical promotion officer, any doctor and others)? (how, why/why not)?
11. Please tell me from your experiences, how do you know about new medicines usages? How do you dispense, recommend and provide new medicines to your patient?
12. How do you deal (e.g. ask about unwell symptoms) when patient ask medicines by name? If patient choice is wrong, how you deal with that?
13. Do you think you are working on drug policy? If no, why? What are the challenges/barriers according to you? Is this impact on provider client interaction? please describe.
14. In conclusion, are there anything else haven’t discussed that you wish to say?

**KII guidelines for Health authority, medical promotion officer, Owner, pharmacy association leader, experience sales person**

**Socio-demographic information about participant:** Age, gender, marital status, number of family members, religion, education, professional degree, training, profession, income (monthly), years of experiences.

**About Drug store/Pharmacy**

1. Please share me how drugs related issues are including in your job responsibilities?
2. In your opinion, what about the health care facilities and practices (structure, access, most uses, advantages, barriers)? Please elaborate (what, when, how and why)?
3. Please tell me, how people become a drug seller in your areas (choice, motivation, training)?
4. Would you please say something about drug seller have any other business or income source parallel with this drug selling? If yes, why?
5. Please say something from your experiences that for what kinds of health concerns people visit at the pharmacy for healthcare and drug purchase? Please elaborate why?
6. Please share with me what drug seller consider as proper treatment and drug attention for clients?
7. Please say something about what the drug seller do if any client refuse to take the services or drug?
8. Please tell me about the profession training (drug store, dispense, primary health) for the seller at pharmacy? Does it impact on client services? If yes/no, please elaborate what, how and why?
9. Would you please share me are there any differences of health/drug services care considering social, cultural, economic and political situation of clients? If yes, please elaborate what, how and why?
10. How does the drug seller perceive about the practices of the patient-centric approach (PCA)? If yes, what, how does they apply PCA knowledge during drug interaction with patients?
11. From your experiences, could you please tell me, for what kinds of drug demand, and health problems usually the drug seller deal with clients?
12. Could you please tell me; what clients usually do (e.g. demand drugs by name, expectation, negotiation, price) when visited to the pharmacy?
13. How does the provider ask clients about their health, drug related problems? Does they talk any greetings, social talk with clients?
14. How do the clients explain about their health, drug related problems to the provider?
15. Could you please tell me from your experiences, do clients have any expectation when they visited to pharmacy? If yes, how do provider and client deal with the expectation?
16. Would you please share, does the provider tells about the necessary information about drug uses (time, dose, course, side effects) during sell?
17. Does the patient listen/follow to providers’ information about drug use?
18. Would you please say about the health information sources for the clients and providers’ (any physicians, medical promotion officer, newspaper, internet, mobile phone)? whom they trust more and why?
19. How does the seller decide about the drug selection for the clients (previous experiences, suggestion from medical promotion officer?
20. Please tell me how do the provider and clients know about new medicines usage? How does the seller dispense, recommend and provide new medicines to patient?
21. How does the seller deal (e.g. ask about unwell symptoms) when patient ask medicines by name? If patient choice is wrong, how the provider deal with that?
22. Could you please share with me any possible gaps related to health/drug care for drug transaction (prescription, non-prescription, self-medication, quality, safety, price, rational use)?
23. What is your opinion about the monitoring, policy action to reduce the patient safety, rational drug usages?
24. In conclusion, are there anything else haven’t discussed that you wish to say?

**Checklist for drug store observation**

**Drug store where sell and purchase drugs**

1. Location of the store (commercial/residential/slum areas)
2. Content of store (privacy room, sitting arrangement, refrigerator, photocopy, mobile recharge, food items)
3. Number of persons involve with selling/prescribing drugs (front desk, counter)
4. Pattern of store (license status, providers’ status e.g. seller/seller cum owner, poorly trained provider/pharmacist)
5. Types of drug sell (Allopathic, Herbal, Ayurveda)
6. Verbal and non-verbal interaction (health complain, drug selection, negotiations, provide information on dose, duration, potential side effects, price)
7. Time spend (waiting, consultation, drug dispense, information giving)
8. Types of everyday practices (e.g. health problems, drugs dispense, sold, generic, branded, health broker, refer to other doctor/clinic/hospital/)
9. Types of client (patient, purchase for family/relatives, economic and education status)
10. Transaction patterns (refer by seller, provider, self-medication, prescription drugs, negotiation)
11. Dealing with medical promotion/information officer, drug monitoring authority.
