# Supplementary material for: The reality of embedded drug purchasing practices: Understanding the sociocultural and economic aspects of the use of medicines in Bangladesh
Source: PLoS One. 2023 Jun 28;18(6):e0287009. doi: 10.1371/journal.pone.0287009 (PMC10306190; doi:10.1371/journal.pone.0287009)
Supplement: Attachment — Submitted filename: PONE-D-22-12723_R2_reviewer.pdf [file pone.0287009.s004.pdf]

PLOS ONE

The Reality of Embedded Drug Purchasing Practices: Understanding the Sociocultural and Economic Aspects of the Use of Medicines in Bangladesh

--Manuscript Draft--

**Manuscript Number:**	PONE-D-22-12723R2
**Article Type:**	Research Article
**Full Title:**	The Reality of Embedded Drug Purchasing Practices: Understanding the Sociocultural and Economic Aspects of the Use of Medicines in Bangladesh
**Short Title:**	Drug transaction and use of medicine at community pharmacy in Bangladesh
**Corresponding Author:**	Md. Shahgahan Miah<br>Shahjalal University of Science and Technology<br>Sylhet, Sylhet Division BANGLADESH
**Keywords:**	Drug purchase, Sociocultural, Economic, Medicine use, Bangladesh
**Abstract:**	Background<br>Purchasing drugs with or without prescription from retail drug shops is standard practice in Bangladesh. However, what actually takes place between the drug seller and customer during the transaction is under-researched. This study explores the drug purchasing practices which underlie the socio-cultural and economic aspects of a Bangladeshi city.<br><br>Methods<br>Adopting ethnographic methods, we conducted thirty in-depth interviews (IDIs) with customers, patients, and sales assistants, and ten key informant interviews (KIIs) with drug sellers, experienced sales assistants and medical representatives. A heterogeneous participant was purposively selected from three drug stores. Transcribed data were coded, and thematic analysis was used to analyze the data.<br><br>Results<br>Results revealed that some individuals visited the drug store with fixed ideas about the name, brand, and dose of the drugs they wanted. In contrast, others come without any preconceived ideas, describe their symptoms and negotiate purchases with the expectation of getting well quickly. Cultural beliefs, trust in sellers, and positive previous experience of using medicines influence the drug purchasing behavior, regardless of any preconceived ideas concerning brand name, and dose. Furthermore, customers sought drugs by trade name but the drug seller often offered a generic substitute because selling non-brand drugs is more profitable. Notably, drugs are bought by installment payments and with loans.<br><br>Conclusion<br>Local context of drug purchasing behavior without prescription drugs can harmed consumers’ health and drug resistance. Policymakers, regulators and healthcare professionals might benefit from policy recommendations to deliver practical information on the rational use of medicines to both sellers and customers.
**Order of Authors:**	Md. Shahgahan Miah, Ph.D.
	Penchan Pradubmook Sherer, Ph.D.
	Nithima Sumpradit, Ph.D.
	Luechai Sringernyuang, Ph.D.
**Response to Reviewers:**	Response to Academic Editor<br><br>Comment<br>Journal Requirements:<br>Please review your reference list to ensure that it is complete and correct. If you have cited papers that have been retracted, please include the rationale for doing so in the manuscript text, or remove these references and replace them with relevant current references. Any changes to the reference list should be mentioned in the rebuttal letter

Powered by Editorial Manager® and ProduXion Manager® from Aries Systems Corporation

that accompanies your revised manuscript. If you need to cite a retracted article, indicate the article's retracted status in the References list and also include a citation and full reference for the retraction notice.

Response
Thank you so much for the comment and suggestions. In response to your observation, we conducted an additional check, and the reference has been updated. At this point, it is hopefully complete and correct according to the requirements of the journal. For your kind information, in our last revised manuscript, we included some new articles according to the reviewer's comments. That is why the reference number changed, and we apologize for not mentioning it in our last response and rebuttal letter. Once again, I would like to thank you for your comments and suggestions.

Additional Editor Comments:
Apologies for taking the time to review your paper. Unfortunately, there is a limited number of reviewers who have the expertise and interest to review this paper. We have reviewers who initially agreed but later did not complete the review on time. I reviewed your paper and have following comments:
This is an interesting and important study that investigated the drug purchasing practices which underlie the socio-cultural and economic aspects of a Bangladeshi city. However, there are a few areas in the manuscripts that can be further improved considering my additional comments. I have following minor observations, please consider them while revising it:

Response
Thank you so much for considering our manuscript for your list and for your appreciation. Yes, we tried to address all your comments and observations, which really improved our manuscript.

Comment: 1.
In the first sentence of the abstract, please replace the word 'standard' with 'common'. Please note that purchasing drugs with or without a prescription from retail drug shops is NOT a standard practice in Bangladesh, but it may be a common practice.

Response
Thank you so much for your valuable suggestion. Yes, we replaced it following your suggestion.
Page: 1 Line: 14

Comment:2.
Please add a sentence under the results of the abstract with the number of IDIs, KIIs and observations you have conducted.

Response:
Thank you so much for the comment. We double checked and found that we included the number of IDIs and KIIs in the methods section of the abstract. Yes, we added a sentence about the observation hours that we conducted during our fieldwork. Thanks again for the comment.
Page: 1 Line: 22-23

Comment: 3.
In your first revision, you did not highlight the texts in which you have made changes. Please do so in your next revision.

Response:
Thank you so much for the comment, and my apologies for the mistake. We followed your comments in our revised manuscript.
Comment:
Page: 4. Line 77, please define what it means about 'C' grade pharmacists.

Response:
Thank you so much for the comment. Yes, we included a sentence that explained the types of pharmacists in Bangladesh.
Page: 4 Line: 80-83

Powered by Editorial Manager® and ProduXion Manager® from Aries Systems Corporation

	Comment: 5.<br>Line 190, please check again the time of the opening hours, whether it is 8.00 AM to 10.00 PM?<br><br>Response:<br>Yes, we double-checked and revised the opening hours. All three drug stores we investigated had regular hours of operation ranging from 8 a.m. to 10 p.m. However, they frequently open early and close late depending on the emergency needs of their clients. Thank you so much for the comment.<br>Page: 10 Line: 193-195<br><br>Comment:6.<br>Please confirm whether the study has been approved by any local ethics committee.<br><br>Response:<br>Thank you so much for your comment. When I submitted the data collection request letter approved by the faculty of graduate studies (FGS), Mahidol University addressed it to the civil surgeon (head of health services at the district level). He wanted to check my ethical permission paperwork and rebate my local ethical approval after reviewing the documents, as I had Mahidol University ethical (IRB) approval.
**Additional Information:**	
**Question**	**Response**
**Financial Disclosure**<br><br>Enter a financial disclosure statement that describes the sources of funding for the work included in this submission. Review the submission guidelines for detailed requirements. View published research articles from *PLOS ONE* for specific examples.<br><br>This statement is required for submission and **will appear in the published article** if the submission is accepted. Please make sure it is accurate.	Yes partial for conducting the fieldwork but the funder have no role in study design, data collection and analysis, decision to publish or preparation of the manuscript

**Unfunded studies**<br>Enter: *The author(s) received no specific funding for this work.*<br><br>**Funded studies**<br>Enter a statement with the following details:<br>• Initials of the authors who received each award<br>• Grant numbers awarded to each author<br>• The full name of each funder<br>• URL of each funder website<br>• Did the sponsors or funders play any role in the study design, data collection and analysis, decision to publish, or preparation of the manuscript?<br>• **NO** - Include this sentence at the end of your statement: *The funders had no role in study design, data collection and analysis, decision to publish, or preparation of the manuscript.*<br>• **YES** - Specify the role(s) played.<br><br>* typeset	
**Competing Interests**<br><br>Use the instructions below to enter a competing interest statement for this submission. On behalf of all authors, disclose any competing interests that could be perceived to bias this work—acknowledging all financial support and any other relevant financial or non-financial competing interests.<br><br>This statement is required for submission and **will appear in the published article** if the submission is accepted. Please make sure it is accurate and that any funding sources listed in your Funding Information later in the submission form are also declared in your Financial Disclosure statement.<br><br>View published research articles from *PLOS ONE* for specific examples.	The authors have declared that no competing interests exist

**NO authors have competing interests**<br><br>Enter: *The authors have declared that no competing interests exist.*<br><br>**Authors with competing interests**<br><br>Enter competing interest details beginning with this statement:<br><br>*I have read the journal's policy and the authors of this manuscript have the following competing interests: [insert competing interests here]*<br><br>* typeset	
**Ethics Statement**<br><br>Enter an ethics statement for this submission. This statement is required if the study involved:<br><br>• Human participants<br>• Human specimens or tissue<br>• Vertebrate animals or cephalopods<br>• Vertebrate embryos or tissues<br>• Field research<br><br>Write "N/A" if the submission does not require an ethics statement.<br><br>General guidance is provided below. Consult the submission guidelines for detailed instructions. **Make sure that all information entered here is included in the Methods section of the manuscript.**	Received the ethical appreciation from the Mahidol University Central Institutional Review Board (MU-CIRB), Thailand, the protocol number MU-CIRB 2020/237.3108

Powered by Editorial Manager® and ProduXion Manager® from Aries Systems Corporation

**Format for specific study types**<br><br>**Human Subject Research (involving human participants and/or tissue)**<br>• Give the name of the institutional review board or ethics committee that approved the study<br>• Include the approval number and/or a statement indicating approval of this research<br>• Indicate the form of consent obtained (written/oral) or the reason that consent was not obtained (e.g. the data were analyzed anonymously)<br><br>**Animal Research (involving vertebrate animals, embryos or tissues)**<br>• Provide the name of the Institutional Animal Care and Use Committee (IACUC) or other relevant ethics board that reviewed the study protocol, and indicate whether they approved this research or granted a formal waiver of ethical approval<br>• Include an approval number if one was obtained<br>• If the study involved *non-human primates*, add *additional details* about animal welfare and steps taken to ameliorate suffering<br>• If anesthesia, euthanasia, or any kind of animal sacrifice is part of the study, include briefly which substances and/or methods were applied<br><br>**Field Research**<br><br>Include the following details if this study involves the collection of plant, animal, or other materials from a natural setting:<br>• Field permit number<br>• Name of the institution or relevant body that granted permission	
**Data Availability**<br><br>Authors are required to make all data underlying the findings described fully available, without restriction, and from the time of publication. PLOS allows rare exceptions to address legal and ethical concerns. See the PLOS Data Policy and FAQ for detailed information.	No - some restrictions will apply

A Data Availability Statement describing where the data can be found is required at submission. Your answers to this question constitute the Data Availability Statement and **will be published in the article**, if accepted.<br><br>**Important:** Stating 'data available on request from the author' is not sufficient. If your data are only available upon request, select 'No' for the first question and explain your exceptional situation in the text box.<br><br>Do the authors confirm that all data underlying the findings described in their manuscript are fully available without restriction?	
**Describe where the data may be found in full sentences. If you are copying our sample text, replace any instances of XXX with the appropriate details.**<br><br>• If the data are **held or will be held in a public repository**, include URLs, accession numbers or DOIs. If this information will only be available after acceptance, indicate this by ticking the box below. For example: *All XXX files are available from the XXX database (accession number(s) XXX, XXX.)*.<br>• If the data are all contained **within the manuscript and/or Supporting Information files**, enter the following: *All relevant data are within the manuscript and its Supporting Information files.*<br>• If neither of these applies but you are able to provide **details of access elsewhere**, with or without limitations, please do so. For example:<br><br>*Data cannot be shared publicly because of [XXX]. Data are available from the XXX Institutional Data Access / Ethics Committee (contact via XXX) for researchers who meet the criteria for access to confidential data.*<br><br>*The data underlying the results presented in the study are available from (include the name of the third party*	Primary data cannot be publicly available as data contains some sensitive issues and restrictions regarding participant consent about data share. The interested researcher can reasonably request Mr. Md Jitu Miah (Email: jitusust@gmail.com), Administrative Officer, Department of Anthropology, Shahjalal University of Science and Technology, Sylhet, Bangladesh. Interview guidelines are available in the Supporting Information files.

Powered by Editorial Manager® and ProduXion Manager® from Aries Systems Corporation

and contact information or URL).<br>• This text is appropriate if the data are owned by a third party and authors do not have permission to share the data.<br><br>* typeset	
Additional data availability information:	

Powered by Editorial Manager® and ProduXion Manager® from Aries Systems Corporation

Manuscript Click here to access/download;Manuscript;Revised manuscript_Clear version.docx

The Reality of Embedded Drug Purchasing Practices: Understanding the Sociocultural and Economic Aspects of the Use of Medicines in Bangladesh

Md. Shahgahan Miah[1,2*#ab] Penchan Pradubmook Sherer[1] Nithima Sumpradit[3#c] Luechai Sringernyuang[1]

[1] Faculty of Social Sciences and Humanities, Mahidol University, Salaya, Thailand

[2] Department of Anthropology, Shahjalal University of Science and Technology, Bangladesh.

[3] Medicines Regulation Division, Food and Drug Administration, Ministry of Public Health, Thailand.

***Corresponding Author**

shahgahan-anp@sust.edu

Abstract

Background

Purchasing drugs with or without prescription from retail drug shops is common practice in Bangladesh. However, what actually takes place between the drug seller and customer during the transaction is under-researched. This study explores the drug purchasing practices which underlie the socio-cultural and economic aspects of a Bangladeshi city.

Methods

Adopting ethnographic methods, we conducted thirty in-depth interviews (IDIs) with customers, patients, and sales assistants, and ten key informant interviews (KIIs) with drug sellers, experienced sales assistants and medical representatives. Thirty hours were spent observing drug sellers' and buyers' nonverbal behavior. A heterogeneous participant was purposively selected

from three drug stores. Transcribed data were coded, and thematic analysis was used to analyze the data.

Results

Results revealed that some individuals visited the drug store with fixed ideas about the name, brand, and dose of the drugs they wanted. In contrast, others come without any preconceived ideas, describe their symptoms and negotiate purchases with the expectation of getting well quickly. Cultural beliefs, trust in sellers, and positive previous experience of using medicines influence the drug purchasing behavior, regardless of any preconceived ideas concerning brand name, and dose. Furthermore, customers sought drugs by trade name but the drug seller often offered a generic substitute because selling non-brand drugs is more profitable. Notably, drugs are bought by installment payments and with loans.

Conclusion

Not sure what the author meant here

Local context of drug purchasing behavior without prescription drugs can harmed consumers' health and drug resistance. Policymakers, regulators and healthcare professionals might benefit from policy recommendations to deliver practical information on the rational use of medicines to both sellers and customers.

Keywords: Drug purchase, Sociocultural, Economic, Medicine use, Bangladesh

Introduction

Global drug purchasing patterns and the way drugs are used in response to illness changed significantly with access to services, the availability of medicines, and aggressive competition among the drug firms [1]. The World Health Organization (WHO) reported that resilient healthcare systems are intrinsically linked to improved access to medication by ensuring rational selection, affordable prices, reliable supply systems, and sustainable financing [2]. One-third of the world's population lacks regular access to essential drugs; 50% of whom are in Asia and Africa [3]. In the South-East Asian Region, at least 65 million people are underprivileged in terms of access to essential medicines and vaccines [4]. Moreover, every year, US$ 42 billion is spent on the inappropriate use of drugs globally [5]. The severity of adverse reactions in low-and-middle-income countries (LMICs) was alarming, with 134 million adverse reactions occurring annually, resulting in 2.6 million deaths [6]. Therefore, WHO launched its third Global Patient Safety Challenge to reduce the level of severe, preventable harm related to medication by 50% over the next five years [5].

The pharmacy is the one place where drug purchasing and pharmaceutical procedures are discussed and negotiated by pharmacists and customers [7]. Modern medicines allow the pharmacist to prescribe drugs for primary care [8]. Therefore, the pharmacist can play a crucial role in delivering primary healthcare and the management of medication that contributes to improving health outcomes by providing clinical services, reducing healthcare costs and traveling and waiting times for a consultation [9,10,11]. In addition, the pharmacist can help to educate patients and guide healthcare staff on the use of medication, its efficacy, how long the drugs should be taken and the potential side effects [10,12]. Research revealed that individuals visited the

This line is not clear. Did the author want to mean " used to visit"

pharmacy to purchase drugs, health supplements and seek advice [13]. Patients also prefer to visit the pharmacy because of the availability of credit, flexible opening hours, and discounts [14,15]. In contrast, low priced and expensive brand drugs are dispensed according to the physical appearance of the customer [16]. A recent study observed that 88% of pharmacists recommend and put more effort into selling a generic substitute [17]. Previous studies have focused on the rationale of drug purchasing from the pharmacists' point of view but there is a lack of research from the customers' perspective that is crucial for ensuring the rational use of drugs.

Majority of patients in developing countries like Bangladesh, the nearest and only source of medicines is the retail drug store outside of the home [18]. 115,439 licensed drug shops have been identified while it was suspected there are an equal number selling drugs without a license. The study found that 80% of people visit retail drug shops or poorly trained quack doctors, a high proportion of whom (76%) self-medicate, and that 92% of drug sellers are 'C' grade pharmacists having only three months of training [18,19]. The Bangladesh Pharmacy Council and Bangladesh Chemist and Druggist Association grade pharmacists "A" for bachelor's degrees, "B" for diplomas, and "C" for three months of training. Notably, 95% of drug sellers are not concerned about the possible side effects of drugs that their patients/customers may suffer [14]. There is plenty of opportunity to prescribe, dispense, and take non-essential and unsafe drugs because of the lack of professional education and training, the desire for profit, and because they are easy prey for the drug firms. There is little research into whether the purchase of drugs might be influenced by sociocultural and economic factors that contribute to irrational drug buying. Thus, this study set out to understand the sociocultural and economic aspects of drug purchasing in a drug store setting. It is hoped that our finding will aid policymakers in reinforcing the rational use of medicines in

Where?

Can the author add any reference?

This is the first time, author introduced two new terms. It would be great if author can define what actually meant in this paper by sociocultural and economic aspects. Is there any evidence in research that these two factors influence drug purchasing behavior? Can author include any conceptual framework or refer that how these factors may influence individual behavior while purchasing drugs? What is the hypothesis and how it is grounded on theory?

What are the evidences that irrational drug buying exists?

primary healthcare settings. In addition, data gather in Bangladesh will contribute at the global level to efforts to achieve universal health coverage (UHC) as well as sustainable development goals (SDGs) 3.8 to provide “access to safe, effective, quality and affordable medicines and vaccines for all”

The author did not mention anything about sociocultural and economic aspects rather than general description of qualitative analysis in the methodology. It may be mentioned how the author assessed these two aspects which are the prime focus of this manuscript?

Methods and Materials

The author may clarify if there were any criterion for selecting study site?

Study time and settings

This ethnographic research was conducted between October 2020 and May 2021 in the *Shahjalal Uposohor* area in ward 22 (administrative zone) in the Sylhet Metropolitan areas of Bangladesh. Sylhet city was identified as being densely populated (1045 of people living in per square kilometer) and having the poorest healthcare indicator in Bangladesh [20]. Within the study areas, high-income and low-income groups live in the suburbs and the '*Tero Ratan slum'* respectively. It has a population of 15,799 with a density of 2,074 and 32 healthcare centers with private retail drug shops, drug stores with a doctor’s consulting room and clinics [21]. Following field study techniques of organizing complex research subjects and data, three topics were chosen as collection tools, namely 'places, people, and events, we selected three private retail drug stores to explore the actual drug purchasing practices using interviews and observation data [22].

Study population, sample and data collection techniques

In order to recruits study participants, the author (SM) spent time at the drug store to observe and meet the customers who visited for the purpose of seeking primary healthcare and buying medication. During the first week of fieldwork, the researcher used to wait at the front counter to observe because the sellers would not allow him to sit inside, possibly because there are sensitive

This line is confusing.

issues around the selling and buying of drugs. However, the researchers' previous qualitative research experiences, student identity, and official letter from Mahidol University confirming that they were collecting data for research purposes, helped to build a good relationship with the drug seller and customers. A week later, the sellers voluntarily invited them to sit in and observe some initial conversations with customers. We purposively recruited participants from three-drug stores who were private retailers, primary healthcare providers and customers. Of the various drug sellers, some of were sellers with limited training (three months), some were rural medical practitioners (RMP) cum drug seller, and some were trainee sales assistants. The customers who visited these drug shops did so either for their own health needs or for those of family members and relatives, and they came with or without any preconceived ideas about the name, brand and dose of the medicines they wanted. Before recruiting participants for interview, we made a note of the customer's visits with the nature of their health problem, and whether they were asking for medicines with or without a prescription. The participants were invited for interview at a time convenient to them after observing the interaction which took place when they purchased their drugs. We recruited 40 participants following the principles of sampling parameters of settings, actors, events, and processes in line the research questions and objectives [23]. We carried out a total of thirty in-depth interviews (IDIs) with lay patients, customers, trainee sales assistants, and ten key informant interviews (KIIs) with primary healthcare providers with drug sellers, very experienced sales assistants, medical representatives and regular customers in order to get a deeper understanding and collect contextualized data (Table 1). In addition, it has been noted that carrying out observation helps in understanding the seller's and buyer's actual interaction behaviors when it comes to conducting interviews. The size of the sample was determined by following the principle of data saturation until reaching the point of repetition and nothing (data, theme, and dimension)

Categories of the respondents seemed do not match with the reported table.

new being found [24]. Saturation level was identified by analyzing the data as an iterative process during the fieldwork.

Data collection procedures

We conducted the interviews based on the participants' voluntary participation, availability and choice of a suitable place. Some interviews were conducted in a private area in the drug store, and some took place elsewhere such as at the customer's home or in a park. We explained about our research, the reason for carrying it out and how the findings would be used when recruiting participants. Semi-structured interview guidelines were developed in order to keep the interviews on track which covered a range of issues such as the drugs requested, consultations, dispensing patterns and negotiations over buying drugs. All interviews were recorded using a digital recorder that ran for 45-60 minutes, although two participants did not agree to recording. Observation data captured local cultural drug purchasing behavior and practices which might not be revealed in the interviews. These observational non-verbal interaction data help to compare, justify and triangulate with the data collected in verbal interviews. All the interviews were conducted in Bengali because it was the researcher's and study participants' first language.

Data analysis

Data was transcribed verbatim after carrying out member checking and then translated into English to ensure consistency of meaning. All four authors were involved in generating open codes manually following research objectives independently. Then an inductive process was followed to analyze the data thematically from the emerging patterns of drug purchasing themes and sub-themes and methodologically triangulated in order to understand the uniformities of the data

[25,26]. Thus, drug transactions between the customer and seller, and the medicines used are described and analyzed as a social process.

Ethical Issues

This study has ethical approval from the Mahidol University Central Institutional Review Board (MU-CIRB), Thailand, protocol number MU-CIRB 2020/237.3108. Ethical issues ensured that those taking part are not put at any risk of trouble or finding themselves in a harmful situation from participating in this study. Both verbal and written consent was obtained before the interviews were initiated. A consent form explaining the study's purpose, the participant's rights and guarantee of confidentiality was briefly but carefully read out prior to starting each interview. A pseudonym was used to protect the identity of the participants.

Results

This section sets out the demographic profiles of the study participants (Table 1). The mean age of participants was 40 (SD ±10) for IDIs and 45 (SD ±9) for the KIIs participants. The majority of participants had post-secondary education but participants from slums had only received primary education. All participants, except two, were married. IDIs participants were from a wide range of occupations: eight were labors, five rickshaw pullers, five worked in non-government organizations (NGO), three in the public sector, and five were teachers, whereas all the female participants were homemakers. KIIs participants were directly involved with selling and prescribing drugs and worked in jobs such as healthcare service providers, drug sellers, medical representatives and experienced sales assistants, whereas two were long standing customers who regularly purchased and took medicines.

Table 1. Socio-demographic background of the participants

IDI (n=30)		KII (n=10)	
**Characteristics**			
**Residents**			
Suburb<br><br>*Slums*	**15**<br><br>**15**	**-**	**-**
Participants			
Client	**15**	*Drug seller*	3
Lay patient	**10**	*Medical representative*	3
Trainee sales assistant	**5**	*Sales assistant*	2
		Client	2
**Age (Mean ± SD)**	**40±10**	**45±9**	
**Sex**			
Male	**25**	*Male*	10
Female	**5**	**-**	
**Education**			
0-5	**7**	*6-10*	**2**
6-10	**5**	*11-12*	**3**
11-12	**8**	*13+*	**5**
13+	**10**	**-**	**-**
**Profession**			
Day labor	**8**	*Provider cum seller*	**3**

What is the difference between clients and lay patients? Authors may consider two sides, supply (users) and demand (providers). Other classification is confusing.

Rickshaw puller	**5**	*Medical representative*	**3**
NGO worker	**4**	*Experienced sales assistant*	**2**
Govt. service holder	**3**	*Regular clients*	**2**
Teacher	**5**	-	-
Homemaker	**5**	-	-

Context

This section seemed to be a mix of background, methods, and results in the result section, the author may reorganize it.

We found various types of drug shops in the study areas considering the location, sellers training and the client flow. We purposively selected three drug stores located in three different settings. The first was located on a commercial road side and was operated by a seller with limited training (‘C’ grade seller having three months training). The second was in an apartment building residential area and operated by a rural medical practitioner cum drug seller. The third one was located next to the slum areas and was operated by a sales assistant and a health assistant (working at the community clinic), who often visits in the evening. Those who dispense drugs are described as a ‘drug seller’ because of their poor professional training. The regular opening hours for dispensing drugs at all three drug stores were between 8 a.m. and 10 a.m. In addition, the sellers frequently open early or remain open late for their clients' emergency requests. However, the drug seller visited customers home in an emergency call. Customers mostly visited the drug store without a prescription, had previous experience of self-medication, and consulted the drug seller in order to select medicines. In addition, drug stores in Bangladesh provide other services such as photocopying, mobile phone top ups, and mobile banking like other superstores, as well as dispensing and selling drugs. Poor regulation and monitoring, a shortage of health workers, and a limited budget for healthcare all influenced the way medicines are bought for self-medication.

it is unclear how the authors concluded these findings? Where is the data? How many of the respondents mentioned these aspects? How the authors measured their cultural beliefs?

Customers' drug purchasing behavior was influenced by cultural beliefs about medicine practices, positive previous experiences, trust on seller and social network of the community people. Several drug sellers mentioned that those living in apartment building areas mostly bought prescription and trade name drugs. However, people from the slum areas sought healthcare and purchased medicines on the recommendation of the

It may be but would be strong to say that a drug store being in a affluent area, their clients will be from higher income group. Did the authors analyzed the characteristics of the respondents who visited these drug stores ? There might be several factors for a client to visit a drug store, one of them is drugs availability. Poor people may also visit to drug seller in the affluent area as they might sell drugs which are not available in other place or may be there are other factors, easy to reach, give discounts.

Table 2. The products sold, the custom

Description	Drug store 1	Drug store 2	Drug 3
**Location**	Higher income residential area	Slum areas	Commercial bazaar areas
**Sellers**	Rural medical practitioner	Sales assistant and occasionally seated health assistant	'C' grade pharmacist (3 months training) and sales assistant
**Clients**	Higher economic, educated and service holder	People living with poverty are rickshaw puller, day labor	Higher income, educated, service holder, and people living with poverty
**Common health problem**	Diabetic, blood pressure, heart diseases	Gastric, Pain, Diarrhea, common cold and fever	Diabetic, blood pressure, diarrhea, pain, accident patient, cold, fever
**Selling Products**	Brand drugs, imported medicines	Non-brand, low-safety, allopathic, herbal drugs	Brand, imported, non-brand, low safety, herbal

Drug store ?

In the following sections, we categorized three key themes of drug purchasing interactions: the purchasing practices, sociocultural and economic reasons of medicine tradeoff discussed below.

Practices of drug purchasing

How did author obtained this information?

People visit with preconceived ideas about the name, brand and dose of drugs

Our data revealed that the individual living in the apartment building visited the drug store with certain preconceived ideas about the trade name, brand and dose of the drugs they wanted. Drug purchasing behavior was influenced by previous satisfactory experiences of using these drugs. Data showed that the drug purchasing behavior of individuals, who seek primary healthcare with preconceived ideas, includes asking for particular drugs by trade name and bring their new and their old prescription to show to the seller. In particular, drugs previously prescribed are picked up together with a full course of medicines. One participant stated in IDI:

> "I have been taking these medicines for three years. I feel good and have no complaint regarding this. So, I mostly bought one month's drugs at the same time".

In contrast, data reported that the customer purchases only half and part of medicines recommended in case a new prescription is given, calculating how much to buy and negotiating the cost of the drugs. One customer shared the issue in IDI:

> "Doctor asked me to visit again after a week. If I do not feel better within a week, the doctor might change the list of medicines during the next visit. That will be a huge loss as the medicines already bought can't be returned".

People who visit the drug store without any ideas about medicines they want

Seeking healthcare and drug purchasing behavior among people from the slum is influenced by the customer's individual wishes, previous experiences, and by the fact that they had similar symptoms to friends or family members. Field data reported that the participants, mostly low-income and illiterate customers, asked the seller about which drugs to take by describing their symptoms, and setting the price of the drug for the ailment. Observation revealed:

> "Give me a five takas' gastric drugs"

Data from in-depth interviews revealed that participants knew the use of particular drugs and they regularly used those. Participants often purchase a drug by trade name, even antibiotics. One customer elaborated in IDI:

> "I have always asked for cef3 (azithromycin) for my sons' cough and fever for the last four years. And, they used to get well with this antibiotic. I do not need to ask the drug seller about medicines."

Drug purchased with a loan and by installment payments

Study participants considered the drug store as the poor people's hospital, a place they could easily access, and where they could seek advice and purchase medicine without having to pay consultancy fees and travel costs. Furthermore, participants can purchase drugs here with a loan. At the end of the month, they pay their medicines bill like other household bills. This arrangement increased trust in the seller and the use of drug. One participant stated his experiences in IDI:

> "I just asked for the drug by name and often by ailment, whatever I need. *Tapan* (sales assistant) writes the amount in the loan book. The day I get my salary I paid the drug bill in cash".

In addition, the customer also seeks to buy a package of expensive drugs by installment payments as well as by buying recommended drugs and those for a particular ailment. Most of the low-income participants purchase daily packages of drugs on the way home from the work. Field data reported that there was a mutual understanding about the installment of medicine and the payment between the sellers and clients as clients often do not buy a complete package of medicine at once but rather buy on a daily basis installment with hand cash payment. The following conversation was recorded during observation:

> “I have been suffering from a fever and have pain all over my body” said the customer. The seller calculates a total of 250 takas for a five-day course of drugs at 50 takas a day (around $3 @ 85 takas per USD) using a calculator and asks customers for a decision. The customer replied he would take one-day’s supply and will buy some each day on the way home after finishing work” (Drug store, *Teroraton* areas)

What if the drug is an antibiotic?

In contrast, paying by installment might encourage the irrational use of drugs as the customer stops taking them when he feels better. One provider cum drug seller explained the customers’ behavior:

> “Some customers cannot afford to buy a full course of drugs. Often they stopped buying if they feel better. If I (seller) asked, the customer often replied that they no longer have a problem so they don’t need to take any more medicine.”

Drug sellers put more effort into the substitution of generic drugs to maximize profit

The efforts put into substituting generic drugs emerged as an important factor in the process of purchasing drugs. Participants reported that the seller put more effort into substituting generic medicines when the medicines the customers asked for by trade name were not available. In

particular, medicines are commonly requested and purchased according to the trade name. Therefore, the seller offers alternative pharmaceutical products in order to maximize profit. Even if the customer asked for a specific brand by trade name, the seller will try to convince the customer to be guided by his expertise on drugs. One observation reported the following conversation during the purchasing of drugs:

> Customers asked for *flucox* (antibiotic), the seller dispensed *flucloxacillin*. When the customer refused to buy the substituted medicines, the seller said that “the only difference is the name of the drug, they are the same as two brothers with the same mother."

Data also identified that if customers want to go to another drug store if they do not agree to the alternative offered, the seller asked them to wait and got the desired brand from another drug shop.

Dispensed non-brand and substandard drugs

Some of the drug sellers reported that they dispensed and sole some unregistered (not registered from the drug administration) local drugs in the drug store. The drug seller or sales assistant dispensed non-brand and substandard drugs in order to maximize profit and did not care about the quality of these medicines. For example, drug stores in slum and commercial areas recommended and dispensed these substandard, non-brand drugs to people who visited without any fixed ideas about which drugs they wanted. Customers did not know about the drug’s quality, brand name, and expiry date because of they trusted the seller. One drug seller stated in KII:

> “The medical representative offers more profit and a higher commission on the non-brand products. As we do not take a consultation fee and have no other source of income, except the profit from drug selling, we tried to sell these drugs to the customer who asked us for medicines, especially the rickshaw pullers and daily laborers”.

Expectations and negotiations in purchasing drugs

Customers' expectations of the medicines, how much they cost and whether it is possible to negotiate the price, influenced drug purchasing behavior from the drug store. Data revealed that the customers expect to get well quickly after taking the medicines. One sales assistant explained:

> "Customers just want to get well immediately after taking the medicine."

Besides, the negotiation process between the seller and customers on the cost and dose of the drugs dispensed has an influence on the choice of drugs. One observation found:

> "Customer: asked the seller for medicines for sneezing and a fever. Seller: dispensed a three-day course of antibiotics. But the customer requested a two-day course because of a lack of money. Then, the seller agrees with customer and suggests that he must complete the full course. The customer smiles and replies 'Of course I will."

Drug transaction practices: the pharmacy as a comprehensive primary health care service

Sociocultural aspects

It is the availability of healthcare services and information at drug store that attracts customers. This includes the prescribing and selling of medicines, measuring blood pressure, testing for diabetes, checking for a fever, providing health information sources and referral to a hospital/clinic, visiting the hospital with customers, booking further appointments at physician's consulting room, home delivery services, and acting as a circumcision center (*Sunnah* Muslim ritual). Participants reported that customers, especially older individuals, can avoid visiting the diagnostic center because of the availability of diabetes diagnosis facilities at their nearest drug

shop. These facilities provide rapid testing of fasting blood samples and reduce the need for carers' visits and the cost and time taken to travel to the center. One participant stated:

> "For a fasting blood test, my father has to wait for hours at the diagnostic center in a long queue, and he is used to having his breakfast early in the morning. Now, he can just give the sample to the nearest pharmacy, get the result, and can have his breakfast at home".

Our research data also reported that many older people and pregnant women who are prescribed medication by the physician that has to be administered by injection find this challenging and it is risky for them to go to the hospital. The drug seller is their only option for accessing this treatment. One female customer stated:

> "I was about thirty-five weeks pregnant and my blood test identified a severe iron deficiency. My gynecologist prescribed seven iron injections. My spouse was worried to hear that I would have to travel to the hospital for the injections! Then the doctor assured him that any drug seller could do this. We got our neighborhood pharmacy doctor to do it".

Trust in the seller and the services they provide: the pharmacist as a healthcare consultant

Trust in the drug seller and their services emerged an important reason for purchasing drugs from the drug store. Data revealed that the customer often visited the drug store with no preconceived ideas and asked for medicines by describing their ailment(s). In particular, participants described the drug store as the peoples' mini hospital and/or clinic, *Osuderdokan* (drug store), *daktarerghor* (doctor house), a referrals center for selecting a doctor and hospital with a referral from the drug seller. During observation, the following conversation was reported between a customer and seller:

> “I slipped over on the road two weeks ago and hurt my right elbow. What should I do? **The customer** presents the symptoms while the **drug seller** replied, “You can go for an x-ray. It will cost 200 takas and then go to the doctor”

Furthermore, the drug seller is thought of as a trustworthy mentor for selecting the best possible physicians, clinic, hospital, diagnostic center and also visited the healthcare center with the customers in cases of emergency. A participant explained:

> “My father felt severe chest pain at midnight, and I was worried about where to go? Then my younger brother asked me to call the pharmacy *dakter* (doctor). Farhad *dakter* visited us with his stethoscope and blood pressure instrument. He did not stop at just referring us to hospital but also went there with us, consulted the doctor, and arranged a hospital bed.”

Competition to achieve a reputation for successful treatment

Participants reported that trust in sellers and their services were linked to having a good relationship and good experiences of treatment. In addition, good experiences of treatment in a drug store setting are influenced by the time it takes to get better. Data showed that not only is the seller motivated to cure ailments quickly in order to create goodwill but the patient or parents also want to get better immediately, even if it means taking high doses of antibiotics. One drug seller explained:

> “If I do not give antibiotics, the patient will not get better quickly because we cannot diagnose diseases. Antibiotics work for any kind of illness although it may harm your health”.

One medical representative shared the fact that drug sellers competed among themselves to build a good reputation in the community that would help to maintain their drug business.

> "There is competition to create a good reputation for curing disease as quickly as possible. They do not think about the side effects of the drugs, and even the buyer wants to get well quickly. There is competition to provide antibiotics in every case."

Discussion

To the best of our knowledge, this is the first qualitative study aimed at understanding the patterns and underlying aspects of drug purchasing practices in a drug store setting in Bangladesh. We have identified some new aspects of drug purchasing patterns in drug stores and in the way people seek primary healthcare from the drug seller and purchase drugs. Beyond this we have looked at the sociocultural and economic aspects of these transactions. Sociodemographic characteristics such as income, education and occupation, together with cultural beliefs and social networks, determine the drug purchasing behavior of buying drugs with brand names whereas the drug seller and sales assistant recommended and dispensed non-brand and substandard drugs to the low income group. We found similar evidence that there are plenty of unlicensed drug stores with the poorly trained drug seller prescribing drugs [8,18,19]. Findings also showed that people negotiate for medicine having a fixed idea of the trade name, brand and dose of the drugs they want, and ask for drugs according to cost and ailment, bring old samples to show to the seller, and discuss what the medicines will treat before deciding what to buy. We have also identified a new trend in purchasing prescription drugs for self-medication, such as buying a half course or partial course of drugs to see whether or not their health improves. In contrast, the drug seller tries to substitute medicines, whether the individual comes with preconceived ideas or has no ideas about drugs they want. Drug

This is not well justified in this study how these linked with economic aspects. This should be clear in the method and grounded on any theory.

I am not sure if this is really a new aspect or regular practice of people with from low socioeconomic group.

sellers even collect the desired drugs from another drug store if they do not have the drugs demanded. Without doubt this was to maximize their profit. Similar findings were reported in earlier studies [17]. The above findings are essential to understanding the misguided purchasing and misuse of drugs.

The sociocultural aspects of buying drugs in the drug store includes the availability of information and services on your doorstep that save the time and cost of travelling, and consultancy fees, as reported by other studies [9,10,11]. This study explores similar findings to those in other studies carried out in Bangladesh that the nearest and only source of medicines is the pharmacy.[18] In addition, our findings reported that, without doubt, the reason for seeking medical care and the medicine purchasing practices was the proximity of services which attract people in the community, not only to the drug store but also to the drug seller who will go to the patient’s home. For instance, the drug seller often made home calls to the elderly and pregnant women to carry out check-ups and give injections and saline infusions. In addition, drug sellers played the role of health educator having recommended physicians, hospitals, and clinics to those worried about their health after listening to their symptoms, but they did not provide information on the efficacy, duration, and potential side effects as suggested by previous studies carried out in developed counties [10,12]. Familiarity with the pharmacist, good experience of treatment and satisfaction with their services creates trust in the provider and their services and this, in turn, influences the seeking of care and purchasing of drugs. For example, curing an ailment immediately creates a good reputation for having a special ability to treat illnesses. In particular, there was competition among the drug sellers to treat patients with a high dose of antibiotics in every case, with the aim of increasing their popularity in the community and thereby selling more drugs. In contrast,

patients and/or parents often avoid getting a diagnosis of their disease and use a course of well-tried antibiotics as ‘powerful’ medicines which can treat a wide range of ailments. These findings will assist policymakers in formulating policy and multilayer interventions to control and ensure the safe use of good quality medicines.

The author may also discuss the about the drug policy of Bangladesh and specifically where this study can contribute.

We found similar findings of patient behavior for visiting the medicine shop due to the availability of credit, flexible opening hours, and discounts on the price [14,15]. However, the practice of purchasing drugs with a loan and by installment payments is a new aspect of drug buying which has increased access to medicines, especially for the underprivileged. However, there is some risk of misuse of drugs because providers and seller are poorly trained and there is a lack of awareness among customers. For example, a customer will discontinue taking the antibiotics when they feel better, which may lead to antibiotic resistance and the misuse of drugs [14]. In addition, the drug sellers are not concerned about customers when it comes to the possible side effects of drugs. These findings about the beliefs, culture and experiences of practices relating to the use of medicines will increase access to medicines with patients being aware of the proper use of drugs and their possible side effects, as well as improving the quality and safety of drugs. In the context of the shortage of health workers, both patients and pharmacists will be in a position to adopt practices that improve primary healthcare in Bangladesh.

This is not clear how the patients will be aware from this findings? Should policymakers take initiatives to aware the patients?

The Limitations of the Study

One limitation of our study was the time constraints as we followed the purposive sampling strategy. Although, a purposive sampling strategy identified rich and informative participants, building up a rapport and becoming familiar with the community was time consuming. Another

limitation was that the study was conducted in urban areas, but healthcare beliefs, seeking medical care and medicine purchasing practices might vary widely in the context of rural areas. Furthermore, sensitivity about the whole issue of recommending, dispensing and selling drugs meant that it was difficult for the sellers to allow us, as outsiders, into their professional space, and observe the services provided at the drug store. The risk was that this might affect the customer's trust. However, the reality of health and drug related practices are context bound, and so these findings can be applied to other places of drug purchasing.

The author may mention theoretical aspects related to healthcare belief and how it is influenced such as using health belief model or revise related terminologies.

Conclusion

This study's findings argue that seeking primary healthcare and purchasing drugs from a drug store is common practice in Bangladesh. The licensed status of the drug store and the sellers' level of education and training, as well as the customer's education, income, cultural beliefs and social network made purchasing drugs a complex issue. Drug purchasing behavior is context-specific because of the individual customer' socio-cultural beliefs, trust in the seller and previous experiences, as well as the financial aspects such as buying drugs with a loan or by installment payments, and avoiding consultancy fees. Individuals buy medicines based on their needs, either with a prescription or for self-medication, by purchasing part or half of a course of medication and do not necessarily complete the recommended course of treatment. Moreover, the way customers self-medicate is influenced by previous experiences, the sellers' positive reputation, and their personal relationship with the seller. In contrast, the drug seller puts more effort into selling generic drug substitutes. These actions might be considered as the unlawful and irrational selling of drugs from the legal and pharmaceutical points of view but customers considered that their behavior was rational. The findings suggest that individuals can access primary care and medicines, especially

those from the low-income group. Bearing in mind existing drug policy, both the customers and sellers are violating the rules of drug selling and buying. Based on the evidence gathered, we conclude the key point is that monitoring and regulation alone is not enough to ensure the supply of safe, good quality medicines and the rational use of these medicines. Thus, the local context of customers' drug practices should be considered in order to ensure the rational use of medicines and a sustainable healthcare system and services.

This seemed not clear, what author exactly want to mean or suggest?

Supporting information

S1. Research tools In-depth Interview and Key Informant Interview Guidelines, Observation checklist

Acknowledgements

We would like acknowledges our study participants for their volunteer participations.

Author Contributions

Conceptualization: Dr. Md. Shahgahan Miah, Dr. Luechai Sringernyuang

Data curation: Dr. Md. Shahgahan Miah

Formal analysis: Dr. Md. Shahgahan Miah, Dr. Penchan Pradubmook Sherer Dr. Nithima Sumpradit Dr. Luechai Sringernyuang

Investigation: Dr. Md. Shahgahan Miah

Methodology: Dr. Md. Shahgahan Miah, Dr. Luechai Sringernyuang, Dr. Penchan Pradubmook Sherer Dr. Nithima Sumpradit

Supervision: Dr. Md. Shahgahan Miah, Dr. Luechai Sringernyuang

Validation: Dr. Md. Shahgahan Miah, Dr. Penchan Pradubmook Sherer Dr. Nithima Sumpradit, Dr. Luechai Sringernyuang

Visualization: Dr. Md. Shahgahan Miah

Writing (original): Dr. Md. Shahgahan Miah, Dr. Luechai Sringernyuang

References

1. Seeberg J. Connecting pills and people: an ethnography of the pharmaceutical nexus in Odisha, India. Med Anthropol Q. 2012;26(2):182-200. doi:10.1111/j.1548-387.2012. 01200.x

2. World Health Organization. Meeting Report: Technical Definitions of Shortages and Stockouts of Medicines and Vaccines: WHO;2016 [Available from: https://www.who.int/medicines/areas/access/Meeting_report_October_Shortages.pdf?ua=1]

3. World Health Organization. WHO medicines strategy 2004–2007.WHO;2004 [Available from: http://whqlibdoc.who.int/hq/2004/WHO_EDM_2004.5.pdf]

4. World Health Organization. Regional Office for South-East Asia. Improving access to medicines in the South-East Asia Region: progress, challenges, priorities. WHO; 2017. [Available from: https://apps.who.int/iris/handle/10665/258750]

5. World Health Organization. WHO launches global eff ort to halve medication-related errors in 5 years. WHO;2017. [Available from: http://www.who.int/mediacentre/news/releases/2017/medication-related-errors/en/]

6. World Health Organization. Patient Safety Fact File. Patient Safety and Risk Management Service Delivery and Safety. WHO; 2019. [Available from: https://apps.who.int/gb/ebwha/pdf_files/WHA72/A72_26-en.pdf]

7. Hardon A, Sanabria, E. Fluid Drugs: Revisiting the Anthropology of Pharmaceuticals. Annu. Rev. Anthropol. 2017; 46:117–132.

8. Shehnaz SI, Agarwal AK, Khan N. A systematic review of self-medication practices among adolescents. J Adolesc Health. 2014;55: 467-483.

9. Waring J, Latif A, Boyd M, Barber N, Elliott R. Pastoral power in the community pharmacy: A Foucauldian analysis of services to promote patient adherence to new medicine use. Social Science & Medicine. 2016;148: 123-130.

10. Sakeena MH, Bennett AA, McLachlan AJ. Enhancing pharmacists' role in developing countries to overcome the challenge of antimicrobial resistance: a narrative review. Antimicrobial Resistance and Infection Control. 2018; 7:63.

11. Bennadi D. Self-medication: A current challenge. Journal of basic and clinical pharmacy. 2013; 5:19-23.

12. Murad MS, Spiers JA, Guirguis LM. Expressing and negotiating face in community pharmacist-patient interactions. Res Social Adm Pharm. 2017; 13:1110-1126.

13. Chua SS, Lim KP, Lee HG. Utilisation of community pharmacists by the general public in Malaysia. Int J Pharm Pract. 2013; 21: 66-69.

14. Saha S, Hossain, MT. Evaluation of medicines dispensing pattern of private pharmacies in Rajshahi, Bangladesh. BMC Health Serv Res. 2017;17:136

15. Khan MU, Khan AN, Ahmed FR, Feroz Z, et al. Patients' opinion of pharmacists and their roles in health care system in Pakistan. J Young Pharm. 2013;5: 90-94.

16. Albadr Y, Khan TM. Factors influencing community pharmacist decision to dispense generic or branded medicines; Eastern Province, Alahsa, Saudi Arabia. Saudi Pharm J.2015; 23:143-146.

17. Al Hussaini M, Alsaffar N, Abdulraheem A. Exploring community pharmacists' knowledge, perception and experiences towards branded and generic medicines in Kuwait: Highlighting the role of pharmacist. Bulletin of Faculty of Pharmacy, Cairo University. 2018; 56: 109-114.
18. Ahmed SM, Naher N, Hossain T, Rawal, LB. Exploring the status of retail private drug shops in Bangladesh and action points for developing an accredited drug shop model: a facility based cross-sectional study. J Pharm Policy Pract. 2017;10: 21.
19. Rakib A, Sarwar MS, Zannah S, Khanum S, Rashid M. A Survey of the Role of Community Pharmacists in Dhaka city, Bangladesh. Bangladesh Pharmaceutical Journal. 2015; 18:137-141.
20. Adams A, Ahmed T, Islam R, Rizvi S, Zakaria R. Mapping health facilities in Sylhet City Corporation, Bangladesh GIZ Health Sector Addressing Bangladesh's Demographic Challenges.2014.
21. Urban Health Atlas. Sylhet City Corporation. Accessed on March 03, 2022. http://urbanhealthatlas.com/maps/city/sylhet#
22. Zaman S. Native among the Natives: Physician Anthropologist Doing Hospital Ethnography at Home. Journal of Contemporary Ethnography. 2008; 37:135-154.
23. Punch KF. Introduction to Social Research: Quantitative & Qualitative Approaches. Sage Publications. 1998
24. Guest G, Bunce A, Johnson L. How many interviews are enough? Experiments in data saturation and variability. Field Methods. 2006; 18:59–82.
25. Braun V, Clarke V. Using thematic analysis in psychology. Qualitative research in psychology. 2006; 3:77–101.

26. Kabir A, Roy S, Begum K, Kabir AH, Miah MS. Factors influencing sanitation and hygiene practices among students in a public university in Bangladesh. PLoS One. 2021;16(9):e0257663. doi: 10.1371/journal.pone.0257663

Click here to access/download
Supporting Information
S1 Interview guidelines.docx

Click here to access/download
Supporting Information
S1-Table-1.tiff

Click here to access/download
Supporting Information
S2-Table-2.tiff

The Reality of Embedded Drug Purchasing Practices: Understanding the Sociocultural and Economic Aspects of the Use of Medicines in Bangladesh

Md. Shahgahan Miah[1,2*#ab] Penchan Pradubmook Sherer[1] Nithima Sumpradit[3#c] Luechai Sringernyuang[1]

[1] Faculty of Social Sciences and Humanities, Mahidol University, Salaya, Thailand

[2] Department of Anthropology, Shahjalal University of Science and Technology, Bangladesh.

[3] Medicines Regulation Division, Food and Drug Administration, Ministry of Public Health, Thailand.

***Corresponding Author**

shahgahan-anp@sust.edu

Abstract

Background

Purchasing drugs with or without prescription from retail drug shops is common practice in Bangladesh. However, what actually takes place between the drug seller and customer during the transaction is under-researched. This study explores the drug purchasing practices which underlie the socio-cultural and economic aspects of a Bangladeshi city.

Methods

Adopting ethnographic methods, we conducted thirty in-depth interviews (IDIs) with customers, patients, and sales assistants, and ten key informant interviews (KIIs) with drug sellers, experienced sales assistants and medical representatives. Thirty hours were spent observing drug sellers' and buyers' nonverbal behavior. A heterogeneous participant was purposively selected

from three drug stores. Transcribed data were coded, and thematic analysis was used to analyze the data.

Results

Results revealed that some individuals visited the drug store with fixed ideas about the name, brand, and dose of the drugs they wanted. In contrast, others come without any preconceived ideas, describe their symptoms and negotiate purchases with the expectation of getting well quickly. Cultural beliefs, trust in sellers, and positive previous experience of using medicines influence the drug purchasing behavior, regardless of any preconceived ideas concerning brand name, and dose. Furthermore, customers sought drugs by trade name but the drug seller often offered a generic substitute because selling non-brand drugs is more profitable. Notably, drugs are bought by installment payments and with loans.

Conclusion

Local context of drug purchasing behavior without prescription drugs can harmed consumers' health and drug resistance. Policymakers, regulators and healthcare professionals might benefit from policy recommendations to deliver practical information on the rational use of medicines to both sellers and customers.

Keywords: Drug purchase, Sociocultural, Economic, Medicine use, Bangladesh

Introduction

Global drug purchasing patterns and the way drugs are used in response to illness changed significantly with access to services, the availability of medicines, and aggressive competition among the drug firms [1]. The World Health Organization (WHO) reported that resilient healthcare systems are intrinsically linked to improved access to medication by ensuring rational selection, affordable prices, reliable supply systems, and sustainable financing [2]. One-third of the world's population lacks regular access to essential drugs; 50% of whom are in Asia and Africa [3]. In the South-East Asian Region, at least 65 million people are underprivileged in terms of access to essential medicines and vaccines [4]. Moreover, every year, US$ 42 billion is spent on the inappropriate use of drugs globally [5]. The severity of adverse reactions in low-and-middle-income countries (LMICs) was alarming, with 134 million adverse reactions occurring annually, resulting in 2.6 million deaths [6]. Therefore, WHO launched its third Global Patient Safety Challenge to reduce the level of severe, preventable harm related to medication by 50% over the next five years [5].

The pharmacy is the one place where drug purchasing and pharmaceutical procedures are discussed and negotiated by pharmacists and customers [7]. Modern medicines allow the pharmacist to prescribe drugs for primary care [8]. Therefore, the pharmacist can play a crucial role in delivering primary healthcare and the management of medication that contributes to improving health outcomes by providing clinical services, reducing healthcare costs and traveling and waiting times for a consultation [9,10,11]. In addition, the pharmacist can help to educate patients and guide healthcare staff on the use of medication, its efficacy, how long the drugs should be taken and the potential side effects [10,12]. Research revealed that individuals visited the

pharmacy to purchase drugs, health supplements and seek advice [13]. Patients also prefer to visit the pharmacy because of the availability of credit, flexible opening hours, and discounts [14,15]. In contrast, low priced and expensive brand drugs are dispensed according to the physical appearance of the customer [16]. A recent study observed that 88% of pharmacists recommend and put more effort into selling a generic substitute [17]. Previous studies have focused on the rationale of drug purchasing from the pharmacists' point of view but there is a lack of research from the customers' perspective that is crucial for ensuring the rational use of drugs.

Majority of patients in developing countries like Bangladesh, the nearest and only source of medicines is the retail drug store outside of the home [18]. 115,439 licensed drug shops have been identified while it was suspected there are an equal number selling drugs without a license. The study found that 80% of people visit retail drug shops or poorly trained quack doctors, a high proportion of whom (76%) self-medicate, and that 92% of drug sellers are 'C' grade pharmacists having only three months of training [18,19]. The Bangladesh Pharmacy Council and Bangladesh Chemist and Druggist Association grade pharmacists "A" for bachelor's degrees, "B" for diplomas, and "C" for three months of training. Notably, 95% of drug sellers are not concerned about the possible side effects of drugs that their patients/customers may suffer [14]. There is plenty of opportunity to prescribe, dispense, and take non-essential and unsafe drugs because of the lack of professional education and training, the desire for profit, and because they are easy prey for the drug firms. There is little research into whether the purchase of drugs might be influenced by sociocultural and economic factors that contribute to irrational drug buying. Thus, this study set out to understand the sociocultural and economic aspects of drug purchasing in a drug store setting. It is hoped that our finding will aid policymakers in reinforcing the rational use of medicines in

primary healthcare settings. In addition, data gather in Bangladesh will contribute at the global level to efforts to achieve universal health coverage (UHC) as well as sustainable development goals (SDGs) 3.8 to provide “access to safe, effective, quality and affordable medicines and vaccines for all”

Methods and Materials

Study time and settings

This ethnographic research was conducted between October 2020 and May 2021 in the *Shahjalal Uposohor* area in ward 22 (administrative zone) in the Sylhet Metropolitan areas of Bangladesh. Sylhet city was identified as being densely populated (1045 of people living in per square kilometer) and having the poorest healthcare indicator in Bangladesh [20]. Within the study areas, high-income and low-income groups live in the suburbs and the '*Tero Ratan slum'* respectively. It has a population of 15,799 with a density of 2,074 and 32 healthcare centers with private retail drug shops, drug stores with a doctor’s consulting room and clinics [21]. Following field study techniques of organizing complex research subjects and data, three topics were chosen as collection tools, namely 'places, people, and events, we selected three private retail drug stores to explore the actual drug purchasing practices using interviews and observation data [22].

Study population, sample and data collection techniques

In order to recruits study participants, the author (SM) spent time at the drug store to observe and meet the customers who visited for the purpose of seeking primary healthcare and buying medication. During the first week of fieldwork, the researcher used to wait at the front counter to observe because the sellers would not allow him to sit inside, possibly because there are sensitive

issues around the selling and buying of drugs. However, the researchers' previous qualitative research experiences, student identity, and official letter from Mahidol University confirming that they were collecting data for research purposes, helped to build a good relationship with the drug seller and customers. A week later, the sellers voluntarily invited them to sit in and observe some initial conversations with customers. We purposively recruited participants from three-drug stores who were private retailers, primary healthcare providers and customers. Of the various drug sellers, some of were sellers with limited training (three months), some were rural medical practitioners (RMP) cum drug seller, and some were trainee sales assistants. The customers who visited these drug shops did so either for their own health needs or for those of family members and relatives, and they came with or without any preconceived ideas about the name, brand and dose of the medicines they wanted. Before recruiting participants for interview, we made a note of the customer's visits with the nature of their health problem, and whether they were asking for medicines with or without a prescription. The participants were invited for interview at a time convenient to them after observing the interaction which took place when they purchased their drugs. We recruited 40 participants following the principles of sampling parameters of settings, actors, events, and processes in line the research questions and objectives [23]. We carried out a total of thirty in-depth interviews (IDIs) with lay patients, customers, trainee sales assistants, and ten key informant interviews (KIIs) with primary healthcare providers with drug sellers, very experienced sales assistants, medical representatives and regular customers in order to get a deeper understanding and collect contextualized data (Table 1). In addition, it has been noted that carrying out observation helps in understanding the seller's and buyer's actual interaction behaviors when it comes to conducting interviews. The size of the sample was determined by following the principle of data saturation until reaching the point of repetition and nothing (data, theme, and dimension)

new being found [24]. Saturation level was identified by analyzing the data as an iterative process during the fieldwork.

Data collection procedures

We conducted the interviews based on the participants' voluntary participation, availability and choice of a suitable place. Some interviews were conducted in a private area in the drug store, and some took place elsewhere such as at the customer's home or in a park.   We explained about our research, the reason for carrying it out and how the findings would be used when recruiting participants. Semi-structured interview guidelines were developed in order to keep the interviews on track which covered a range of issues such as the drugs requested, consultations, dispensing patterns and negotiations over buying drugs. All interviews were recorded using a digital recorder that ran for 45-60 minutes, although two participants did not agree to recording. Observation data captured local cultural drug purchasing behavior and practices which might not be revealed in the interviews. These observational non-verbal interaction data help to compare, justify and triangulate with the data collected in verbal interviews. All the interviews were conducted in Bengali because it was the researcher's and study participants' first language.

Data analysis

Data was transcribed verbatim after carrying out member checking and then translated into English to ensure consistency of meaning. All four authors were involved in generating open codes manually following research objectives independently. Then an inductive process was followed to analyze the data thematically from the emerging patterns of drug purchasing themes and sub-themes and methodologically triangulated in order to understand the uniformities of the data

[25,26]. Thus, drug transactions between the customer and seller, and the medicines used are described and analyzed as a social process.

Ethical Issues

This study has ethical approval from the Mahidol University Central Institutional Review Board (MU-CIRB), Thailand, protocol number MU-CIRB 2020/237.3108. Ethical issues ensured that those taking part are not put at any risk of trouble or finding themselves in a harmful situation from participating in this study. Both verbal and written consent was obtained before the interviews were initiated. A consent form explaining the study's purpose, the participant's rights and guarantee of confidentiality was briefly but carefully read out prior to starting each interview. A pseudonym was used to protect the identity of the participants.

Results

This section sets out the demographic profiles of the study participants (Table 1). The mean age of participants was 40 (SD ±10) for IDIs and 45 (SD ±9) for the KIIs participants. The majority of participants had post-secondary education but participants from slums had only received primary education. All participants, except two, were married. IDIs participants were from a wide range of occupations: eight were labors, five rickshaw pullers, five worked in non-government organizations (NGO), three in the public sector, and five were teachers, whereas all the female participants were homemakers. KIIs participants were directly involved with selling and prescribing drugs and worked in jobs such as healthcare service providers, drug sellers, medical representatives and experienced sales assistants, whereas two were long standing customers who regularly purchased and took medicines.

Table 1. Socio-demographic background of the participants

IDI (n=30)		KII (n=10)	
**Characteristics**			
**Residents**			
Suburb<br>*Slums*	**15**<br>**15**	**-**	**-**
Participants			
Client	**15**	*Drug seller*	3
Lay patient	**10**	*Medical representative*	3
Trainee sales assistant	**5**	*Sales assistant*	2
		Client	2
**Age (Mean ± SD)**	**40±10**	**45±9**	
**Sex**			
Male	**25**	*Male*	10
Female	**5**	**-**	
**Education**			
0-5	**7**	*6-10*	**2**
6-10	**5**	*11-12*	**3**
11-12	**8**	*13+*	**5**
13+	**10**	**-**	**-**
**Profession**			
Day labor	**8**	*Provider cum seller*	**3**

Rickshaw puller	**5**	*Medical representative*	**3**
NGO worker	**4**	*Experienced sales assistant*	**2**
Govt. service holder	**3**	*Regular clients*	**2**
Teacher	**5**	-	**-**
Homemaker	**5**	-	**-**

Context

We found various types of drug shops in the study areas considering the location, sellers training and the client flow. We purposively selected three drug stores located in three different settings. The first was located on a commercial road side and was operated by a seller with limited training ('C' grade seller having three months training). The second was in an apartment building residential area and operated by a rural medical practitioner cum drug seller. The third one was located next to the slum areas and was operated by a sales assistant and a health assistant (working at the community clinic), who often visits in the evening. Those who dispense drugs are described as a 'drug seller' because of their poor professional training. The regular opening hours for dispensing drugs at all three drug stores were between 8 a.m. and 10 a.m. In addition, the sellers frequently open early or remain open late for their clients' emergency requests. However, the drug seller visited customers home in an emergency call. Customers mostly visited the drug store without a prescription, had previous experience of self-medication, and consulted the drug seller in order to select medicines. In addition, drug stores in Bangladesh provide other services such as photocopying, mobile phone top ups, and mobile banking like other superstores, as well as dispensing and selling drugs. Poor regulation and monitoring, a shortage of health workers, and a limited budget for healthcare all influenced the way medicines are bought for self-medication.

Customers' drug purchasing behavior was influenced by cultural beliefs about medicine practices, positive previous experiences, trust on seller and social network of the community people. Several drug sellers mentioned that those living in apartment building areas mostly bought prescription and trade name drugs. However, people from the slum areas sought healthcare and purchased medicines on the recommendation of the sellers.

Table 2. The products sold, the customers, and the range of drug purchased

Description	Drug store 1	Drug store 2	Drug 3
**Location**	Higher income residential area	Slum areas	Commercial bazaar areas
**Sellers**	Rural medical practitioner	Sales assistant and occasionally seated health assistant	'C' grade pharmacist (3 months training) and sales assistant
**Clients**	Higher economic, educated and service holder	People living with poverty are rickshaw puller, day labor	Higher income, educated, service holder, and people living with poverty
**Common health problem**	Diabetic, blood pressure, heart diseases	Gastric, Pain, Diarrhea, common cold and fever	Diabetic, blood pressure, diarrhea, pain, accident patient, cold, fever
**Selling Products**	Brand drugs, imported medicines	Non-brand, low-safety, allopathic, herbal drugs	Brand, imported, non-brand, low safety, herbal

In the following sections, we categorized three key themes of drug purchasing interactions: the purchasing practices, sociocultural and economic reasons of medicine tradeoff discussed below.

Practices of drug purchasing

People visit with preconceived ideas about the name, brand and dose of drugs

Our data revealed that the individual living in the apartment building visited the drug store with certain preconceived ideas about the trade name, brand and dose of the drugs they wanted. Drug purchasing behavior was influenced by previous satisfactory experiences of using these drugs. Data showed that the drug purchasing behavior of individuals, who seek primary healthcare with preconceived ideas, includes asking for particular drugs by trade name and bring their new and their old prescription to show to the seller. In particular, drugs previously prescribed are picked up together with a full course of medicines. One participant stated in IDI:

> "I have been taking these medicines for three years. I feel good and have no complaint regarding this. So, I mostly bought one month's drugs at the same time".

In contrast, data reported that the customer purchases only half and part of medicines recommended in case a new prescription is given, calculating how much to buy and negotiating the cost of the drugs. One customer shared the issue in IDI:

> "Doctor asked me to visit again after a week. If I do not feel better within a week, the doctor might change the list of medicines during the next visit. That will be a huge loss as the medicines already bought can't be returned".

People who visit the drug store without any ideas about medicines they want

Seeking healthcare and drug purchasing behavior among people from the slum is influenced by the customer's individual wishes, previous experiences, and by the fact that they had similar symptoms to friends or family members. Field data reported that the participants, mostly low-income and illiterate customers, asked the seller about which drugs to take by describing their symptoms, and setting the price of the drug for the ailment. Observation revealed:

> "Give me a five takas' gastric drugs"

Data from in-depth interviews revealed that participants knew the use of particular drugs and they regularly used those. Participants often purchase a drug by trade name, even antibiotics. One customer elaborated in IDI:

> "I have always asked for cef3 (azithromycin) for my sons' cough and fever for the last four years. And, they used to get well with this antibiotic. I do not need to ask the drug seller about medicines."

Drug purchased with a loan and by installment payments

Study participants considered the drug store as the poor people's hospital, a place they could easily access, and where they could seek advice and purchase medicine without having to pay consultancy fees and travel costs. Furthermore, participants can purchase drugs here with a loan. At the end of the month, they pay their medicines bill like other household bills. This arrangement increased trust in the seller and the use of drug. One participant stated his experiences in IDI:

> "I just asked for the drug by name and often by ailment, whatever I need. *Tapan* (sales assistant) writes the amount in the loan book. The day I get my salary I paid the drug bill in cash".

In addition, the customer also seeks to buy a package of expensive drugs by installment payments as well as by buying recommended drugs and those for a particular ailment. Most of the low-income participants purchase daily packages of drugs on the way home from the work. Field data reported that there was a mutual understanding about the installment of medicine and the payment between the sellers and clients as clients often do not buy a complete package of medicine at once but rather buy on a daily basis installment with hand cash payment. The following conversation was recorded during observation:

> "I have been suffering from a fever and have pain all over my body" said the customer. The seller calculates a total of 250 takas for a five-day course of drugs at 50 takas a day (around $3 @ 85 takas per USD) using a calculator and asks customers for a decision. The customer replied he would take one-day's supply and will buy some each day on the way home after finishing work" (Drug store, *Teroraton* areas)

In contrast, paying by installment might encourage the irrational use of drugs as the customer stops taking them when he feels better. One provider cum drug seller explained the customers' behavior:

> "Some customers cannot afford to buy a full course of drugs. Often they stopped buying if they feel better. If I (seller) asked, the customer often replied that they no longer have a problem so they don't need to take any more medicine."

Drug sellers put more effort into the substitution of generic drugs to maximize profit

The efforts put into substituting generic drugs emerged as an important factor in the process of purchasing drugs. Participants reported that the seller put more effort into substituting generic medicines when the medicines the customers asked for by trade name were not available. In

particular, medicines are commonly requested and purchased according to the trade name. Therefore, the seller offers alternative pharmaceutical products in order to maximize profit. Even if the customer asked for a specific brand by trade name, the seller will try to convince the customer to be guided by his expertise on drugs. One observation reported the following conversation during the purchasing of drugs:

> Customers asked for *flucox* (antibiotic), the seller dispensed *flucloxacillin*. When the customer refused to buy the substituted medicines, the seller said that “the only difference is the name of the drug, they are the same as two brothers with the same mother."

Data also identified that if customers want to go to another drug store if they do not agree to the alternative offered, the seller asked them to wait and got the desired brand from another drug shop.

Dispensed non-brand and substandard drugs

Some of the drug sellers reported that they dispensed and sole some unregistered (not registered from the drug administration) local drugs in the drug store. The drug seller or sales assistant dispensed non-brand and substandard drugs in order to maximize profit and did not care about the quality of these medicines. For example, drug stores in slum and commercial areas recommended and dispensed these substandard, non-brand drugs to people who visited without any fixed ideas about which drugs they wanted. Customers did not know about the drug’s quality, brand name, and expiry date because of they trusted the seller. One drug seller stated in KII:

> “The medical representative offers more profit and a higher commission on the non-brand products. As we do not take a consultation fee and have no other source of income, except the profit from drug selling, we tried to sell these drugs to the customer who asked us for medicines, especially the rickshaw pullers and daily laborers”.

Expectations and negotiations in purchasing drugs

Customers' expectations of the medicines, how much they cost and whether it is possible to negotiate the price, influenced drug purchasing behavior from the drug store. Data revealed that the customers expect to get well quickly after taking the medicines. One sales assistant explained:

> "Customers just want to get well immediately after taking the medicine."

Besides, the negotiation process between the seller and customers on the cost and dose of the drugs dispensed has an influence on the choice of drugs. One observation found:

> "Customer: asked the seller for medicines for sneezing and a fever. Seller: dispensed a three-day course of antibiotics. But the customer requested a two-day course because of a lack of money. Then, the seller agrees with customer and suggests that he must complete the full course. The customer smiles and replies 'Of course I will."

Drug transaction practices: the pharmacy as a comprehensive primary health care service

Sociocultural aspects

It is the availability of healthcare services and information at drug store that attracts customers. This includes the prescribing and selling of medicines, measuring blood pressure, testing for diabetes, checking for a fever, providing health information sources and referral to a hospital/clinic, visiting the hospital with customers, booking further appointments at physician's consulting room, home delivery services, and acting as a circumcision center (*Sunnah* Muslim ritual). Participants reported that customers, especially older individuals, can avoid visiting the diagnostic center because of the availability of diabetes diagnosis facilities at their nearest drug

shop. These facilities provide rapid testing of fasting blood samples and reduce the need for carers' visits and the cost and time taken to travel to the center. One participant stated:

> "For a fasting blood test, my father has to wait for hours at the diagnostic center in a long queue, and he is used to having his breakfast early in the morning. Now, he can just give the sample to the nearest pharmacy, get the result, and can have his breakfast at home".

Our research data also reported that many older people and pregnant women who are prescribed medication by the physician that has to be administered by injection find this challenging and it is risky for them to go to the hospital. The drug seller is their only option for accessing this treatment. One female customer stated:

> "I was about thirty-five weeks pregnant and my blood test identified a severe iron deficiency. My gynecologist prescribed seven iron injections. My spouse was worried to hear that I would have to travel to the hospital for the injections! Then the doctor assured him that any drug seller could do this. We got our neighborhood pharmacy doctor to do it".

Trust in the seller and the services they provide: the pharmacist as a healthcare consultant

Trust in the drug seller and their services emerged an important reason for purchasing drugs from the drug store. Data revealed that the customer often visited the drug store with no preconceived ideas and asked for medicines by describing their ailment(s). In particular, participants described the drug store as the peoples' mini hospital and/or clinic, *Osuderdokan* (drug store), *daktarerghor* (doctor house), a referrals center for selecting a doctor and hospital with a referral from the drug seller. During observation, the following conversation was reported between a customer and seller:

> "I slipped over on the road two weeks ago and hurt my right elbow. What should I do? **The customer** presents the symptoms while the **drug** s**eller** replied, "You can go for an x-ray. It will cost 200 takas and then go to the doctor"

Furthermore, the drug seller is thought of as a trustworthy mentor for selecting the best possible physicians, clinic, hospital, diagnostic center and also visited the healthcare center with the customers in cases of emergency. A participant explained:

> "My father felt severe chest pain at midnight, and I was worried about where to go? Then my younger brother asked me to call the pharmacy *dakter* (doctor). Farhad *dakter* visited us with his stethoscope and blood pressure instrument. He did not stop at just referring us to hospital but also went there with us, consulted the doctor, and arranged a hospital bed."

Competition to achieve a reputation for successful treatment

Participants reported that trust in sellers and their services were linked to having a good relationship and good experiences of treatment. In addition, good experiences of treatment in a drug store setting are influenced by the time it takes to get better. Data showed that not only is the seller motivated to cure ailments quickly in order to create goodwill but the patient or parents also want to get better immediately, even if it means taking high doses of antibiotics. One drug seller explained:

> "If I do not give antibiotics, the patient will not get better quickly because we cannot diagnose diseases. Antibiotics work for any kind of illness although it may harm your health".

One medical representative shared the fact that drug sellers competed among themselves to build a good reputation in the community that would help to maintain their drug business.

> "There is competition to create a good reputation for curing disease as quickly as possible. They do not think about the side effects of the drugs, and even the buyer wants to get well quickly. There is competition to provide antibiotics in every case."

Discussion

To the best of our knowledge, this is the first qualitative study aimed at understanding the patterns and underlying aspects of drug purchasing practices in a drug store setting in Bangladesh. We have identified some new aspects of drug purchasing patterns in drug stores and in the way people seek primary healthcare from the drug seller and purchase drugs. Beyond this we have looked at the sociocultural and economic aspects of these transactions. Sociodemographic characteristics such as income, education and occupation, together with cultural beliefs and social networks, determine the drug purchasing behavior of buying drugs with brand names whereas the drug seller and sales assistant recommended and dispensed non-brand and substandard drugs to the low income group. We found similar evidence that there are plenty of unlicensed drug stores with the poorly trained drug seller prescribing drugs [8,18,19]. Findings also showed that people negotiate for medicine having a fixed idea of the trade name, brand and dose of the drugs they want, and ask for drugs according to cost and ailment, bring old samples to show to the seller, and discuss what the medicines will treat before deciding what to buy. We have also identified a new trend in purchasing prescription drugs for self-medication, such as buying a half course or partial course of drugs to see whether or not their health improves. In contrast, the drug seller tries to substitute medicines, whether the individual comes with preconceived ideas or has no ideas about drugs they want. Drug

sellers even collect the desired drugs from another drug store if they do not have the drugs demanded. Without doubt this was to maximize their profit. Similar findings were reported in earlier studies [17]. The above findings are essential to understanding the misguided purchasing and misuse of drugs.

The sociocultural aspects of buying drugs in the drug store includes the availability of information and services on your doorstep that save the time and cost of travelling, and consultancy fees, as reported by other studies [9,10,11]. This study explores similar findings to those in other studies carried out in Bangladesh that the nearest and only source of medicines is the pharmacy.[18] In addition, our findings reported that, without doubt, the reason for seeking medical care and the medicine purchasing practices was the proximity of services which attract people in the community, not only to the drug store but also to the drug seller who will go to the patient's home. For instance, the drug seller often made home calls to the elderly and pregnant women to carry out check-ups and give injections and saline infusions. In addition, drug sellers played the role of health educator having recommended physicians, hospitals, and clinics to those worried about their health after listening to their symptoms, but they did not provide information on the efficacy, duration, and potential side effects as suggested by previous studies carried out in developed counties [10,12]. Familiarity with the pharmacist, good experience of treatment and satisfaction with their services creates trust in the provider and their services and this, in turn, influences the seeking of care and purchasing of drugs. For example, curing an ailment immediately creates a good reputation for having a special ability to treat illnesses. In particular, there was competition among the drug sellers to treat patients with a high dose of antibiotics in every case, with the aim of increasing their popularity in the community and thereby selling more drugs. In contrast,

patients and/or parents often avoid getting a diagnosis of their disease and use a course of well-tried antibiotics as ‘powerful’ medicines which can treat a wide range of ailments. These findings will assist policymakers in formulating policy and multilayer interventions to control and ensure the safe use of good quality medicines.

We found similar findings of patient behavior for visiting the medicine shop due to the availability of credit, flexible opening hours, and discounts on the price [14,15]. However, the practice of purchasing drugs with a loan and by installment payments is a new aspect of drug buying which has increased access to medicines, especially for the underprivileged. However, there is some risk of misuse of drugs because providers and seller are poorly trained and there is a lack of awareness among customers. For example, a customer will discontinue taking the antibiotics when they feel better, which may lead to antibiotic resistance and the misuse of drugs [14]. In addition, the drug sellers are not concerned about customers when it comes to the possible side effects of drugs. These findings about the beliefs, culture and experiences of practices relating to the use of medicines will increase access to medicines with patients being aware of the proper use of drugs and their possible side effects, as well as improving the quality and safety of drugs. In the context of the shortage of health workers, both patients and pharmacists will be in a position to adopt practices that improve primary healthcare in Bangladesh.

The Limitations of the Study

One limitation of our study was the time constraints as we followed the purposive sampling strategy. Although, a purposive sampling strategy identified rich and informative participants, building up a rapport and becoming familiar with the community was time consuming. Another

limitation was that the study was conducted in urban areas, but healthcare beliefs, seeking medical care and medicine purchasing practices might vary widely in the context of rural areas. Furthermore, sensitivity about the whole issue of recommending, dispensing and selling drugs meant that it was difficult for the sellers to allow us, as outsiders, into their professional space, and observe the services provided at the drug store. The risk was that this might affect the customer's trust. However, the reality of health and drug related practices are context bound, and so these findings can be applied to other places of drug purchasing.

Conclusion

This study's findings argue that seeking primary healthcare and purchasing drugs from a drug store is common practice in Bangladesh. The licensed status of the drug store and the sellers' level of education and training, as well as the customer's education, income, cultural beliefs and social network made purchasing drugs a complex issue. Drug purchasing behavior is context-specific because of the individual customer' socio-cultural beliefs, trust in the seller and previous experiences, as well as the financial aspects such as buying drugs with a loan or by installment payments, and avoiding consultancy fees. Individuals buy medicines based on their needs, either with a prescription or for self-medication, by purchasing part or half of a course of medication and do not necessarily complete the recommended course of treatment. Moreover, the way customers self-medicate is influenced by previous experiences, the sellers' positive reputation, and their personal relationship with the seller. In contrast, the drug seller puts more effort into selling generic drug substitutes. These actions might be considered as the unlawful and irrational selling of drugs from the legal and pharmaceutical points of view but customers considered that their behavior was rational. The findings suggest that individuals can access primary care and medicines, especially

those from the low-income group. Bearing in mind existing drug policy, both the customers and sellers are violating the rules of drug selling and buying. Based on the evidence gathered, we conclude the key point is that monitoring and regulation alone is not enough to ensure the supply of safe, good quality medicines and the rational use of these medicines. Thus, the local context of customers' drug practices should be considered in order to ensure the rational use of medicines and a sustainable healthcare system and services.

Supporting information

S1. Research tools In-depth Interview and Key Informant Interview Guidelines, Observation checklist

Acknowledgements

We would like acknowledges our study participants for their volunteer participations.

Author Contributions

Conceptualization: Dr. Md. Shahgahan Miah, Dr. Luechai Sringernyuang

Data curation: Dr. Md. Shahgahan Miah

Formal analysis: Dr. Md. Shahgahan Miah, Dr. Penchan Pradubmook Sherer Dr. Nithima Sumpradit Dr. Luechai Sringernyuang

Investigation: Dr. Md. Shahgahan Miah

Methodology: Dr. Md. Shahgahan Miah, Dr. Luechai Sringernyuang, Dr. Penchan Pradubmook Sherer Dr. Nithima Sumpradit

Supervision: Dr. Md. Shahgahan Miah, Dr. Luechai Sringernyuang

Validation: Dr. Md. Shahgahan Miah, Dr. Penchan Pradubmook Sherer Dr. Nithima Sumpradit, Dr. Luechai Sringernyuang

Visualization: Dr. Md. Shahgahan Miah

Writing (original): Dr. Md. Shahgahan Miah, Dr. Luechai Sringernyuang

References

1. Seeberg J. Connecting pills and people: an ethnography of the pharmaceutical nexus in Odisha, India. Med Anthropol Q. 2012;26(2):182-200. doi:10.1111/j.1548-387.2012. 01200.x

2. World Health Organization. Meeting Report: Technical Definitions of Shortages and Stockouts of Medicines and Vaccines: WHO;2016 [Available from: https://www.who.int/medicines/areas/access/Meeting_report_October_Shortages.pdf?ua=1]

3. World Health Organization. WHO medicines strategy 2004–2007.WHO;2004 [Available from: http://whqlibdoc.who.int/hq/2004/WHO_EDM_2004.5.pdf]

4. World Health Organization. Regional Office for South-East Asia. Improving access to medicines in the South-East Asia Region: progress, challenges, priorities. WHO; 2017. [Available from: https://apps.who.int/iris/handle/10665/258750]

5. World Health Organization. WHO launches global eff ort to halve medication-related errors in 5 years. WHO;2017. [Available from: http://www.who.int/mediacentre/news/releases/2017/medication-related-errors/en/]

6. World Health Organization. Patient Safety Fact File. Patient Safety and Risk Management Service Delivery and Safety. WHO; 2019. [Available from: https://apps.who.int/gb/ebwha/pdf_files/WHA72/A72_26-en.pdf]

7. Hardon A, Sanabria, E. Fluid Drugs: Revisiting the Anthropology of Pharmaceuticals. Annu. Rev. Anthropol. 2017; 46:117–132.

8. Shehnaz SI, Agarwal AK, Khan N. A systematic review of self-medication practices among adolescents. J Adolesc Health. 2014;55: 467-483.

9. Waring J, Latif A, Boyd M, Barber N, Elliott R. Pastoral power in the community pharmacy: A Foucauldian analysis of services to promote patient adherence to new medicine use. Social Science & Medicine. 2016;148: 123-130.

10. Sakeena MH, Bennett AA, McLachlan AJ. Enhancing pharmacists' role in developing countries to overcome the challenge of antimicrobial resistance: a narrative review. Antimicrobial Resistance and Infection Control. 2018; 7:63.

11. Bennadi D. Self-medication: A current challenge. Journal of basic and clinical pharmacy. 2013; 5:19-23.

12. Murad MS, Spiers JA, Guirguis LM. Expressing and negotiating face in community pharmacist-patient interactions. Res Social Adm Pharm. 2017; 13:1110-1126.

13. Chua SS, Lim KP, Lee HG. Utilisation of community pharmacists by the general public in Malaysia. Int J Pharm Pract. 2013; 21: 66-69.

14. Saha S, Hossain, MT. Evaluation of medicines dispensing pattern of private pharmacies in Rajshahi, Bangladesh. BMC Health Serv Res. 2017;17:136

15. Khan MU, Khan AN, Ahmed FR, Feroz Z, et al. Patients' opinion of pharmacists and their roles in health care system in Pakistan. J Young Pharm. 2013;5: 90-94.

16. Albadr Y, Khan TM. Factors influencing community pharmacist decision to dispense generic or branded medicines; Eastern Province, Alahsa, Saudi Arabia. Saudi Pharm J.2015; 23:143-146.

17. Al Hussaini M, Alsaffar N, Abdulraheem A. Exploring community pharmacists' knowledge, perception and experiences towards branded and generic medicines in Kuwait: Highlighting the role of pharmacist. Bulletin of Faculty of Pharmacy, Cairo University. 2018; 56: 109-114.

18. Ahmed SM, Naher N, Hossain T, Rawal, LB. Exploring the status of retail private drug shops in Bangladesh and action points for developing an accredited drug shop model: a facility based cross-sectional study. J Pharm Policy Pract. 2017;10: 21.

19. Rakib A, Sarwar MS, Zannah S, Khanum S, Rashid M. A Survey of the Role of Community Pharmacists in Dhaka city, Bangladesh. Bangladesh Pharmaceutical Journal. 2015; 18:137-141.

20. Adams A, Ahmed T, Islam R, Rizvi S, Zakaria R. Mapping health facilities in Sylhet City Corporation, Bangladesh GIZ Health Sector Addressing Bangladesh's Demographic Challenges.2014.

21. Urban Health Atlas. Sylhet City Corporation. Accessed on March 03, 2022. http://urbanhealthatlas.com/maps/city/sylhet#

22. Zaman S. Native among the Natives: Physician Anthropologist Doing Hospital Ethnography at Home. Journal of Contemporary Ethnography. 2008; 37:135-154.

23. Punch KF. Introduction to Social Research: Quantitative & Qualitative Approaches. Sage Publications. 1998

24. Guest G, Bunce A, Johnson L. How many interviews are enough? Experiments in data saturation and variability. Field Methods. 2006; 18:59–82.

25. Braun V, Clarke V. Using thematic analysis in psychology. Qualitative research in psychology. 2006; 3:77–101.

26. Kabir A, Roy S, Begum K, Kabir AH, Miah MS. Factors influencing sanitation and hygiene practices among students in a public university in Bangladesh. PLoS One. 2021;16(9):e0257663. doi: 10.1371/journal.pone.0257663

Response to Academic Editor

Comment

Journal Requirements:

Please review your reference list to ensure that it is complete and correct. If you have cited papers that have been retracted, please include the rationale for doing so in the manuscript text, or remove these references and replace them with relevant current references. Any changes to the reference list should be mentioned in the rebuttal letter that accompanies your revised manuscript. If you need to cite a retracted article, indicate the article's retracted status in the References list and also include a citation and full reference for the retraction notice.

Response

Thank you so much for the comment and suggestions. In response to your observation, we conducted an additional check, and the reference has been updated. At this point, it is hopefully complete and correct according to the requirements of the journal. For your kind information, in our last revised manuscript, we included some new articles according to the reviewer's comments. That is why the reference number changed, and we apologize for not mentioning it in our last response and rebuttal letter. Once again, I would like to thank you for your comments and suggestions.

Additional Editor Comments:

Apologies for taking the time to review your paper. Unfortunately, there is a limited number of reviewers who have the expertise and interest to review this paper. We have reviewers who initially agreed but later did not complete the review on time. I reviewed your paper and have following comments:

This is an interesting and important study that investigated the drug purchasing practices which underlie the socio-cultural and economic aspects of a Bangladeshi city. However, there are a few areas in the manuscripts that can be further improved considering my additional comments. I have following minor observations, please consider them while revising it:

Response

Thank you so much for considering our manuscript for your list and for your appreciation. Yes, we tried to address all your comments and observations, which really improved our manuscript.

Comment: 1.

In the first sentence of the abstract, please replace the word 'standard' with 'common'. Please note that purchasing drugs with or without a prescription from retail drug shops is NOT a standard practice in Bangladesh, but it may be a common practice.

Response

Thank you so much for your valuable suggestion. Yes, we replaced it following your suggestion.

Page: 1 **Line:** 14

Comment:2.

Please add a sentence under the results of the abstract with the number of IDIs, KIIs and observations you have conducted.

Response:

Thank you so much for the comment. We double checked and found that we included the number of IDIs and KIIs in the methods section of the abstract. Yes, we added a sentence about the observation hours that we conducted during our fieldwork. Thanks again for the comment.

Page: 1 **Line:** 22-23

Comment: 3.

In your first revision, you did not highlight the texts in which you have made changes. Please do so in your next revision.

Response:

Thank you so much for the comment, and my apologies for the mistake. We followed your comments in our revised manuscript.

Comment:

Page: 4. Line 77, please define what it means about ‘C’ grade pharmacists.

Response:

Thank you so much for the comment. Yes, we included a sentence that explained the types of pharmacists in Bangladesh.

Page: 4 **Line:** 80-83

Comment: 5.

Line 190, please check again the time of the opening hours, whether it is 8.00 AM to 10.00 PM?

Response:

Yes, we double-checked and revised the opening hours. All three drug stores we investigated had regular hours of operation ranging from 8 a.m. to 10 p.m. However, they frequently open early and close late depending on the emergency needs of their clients. Thank you so much for the comment.

Page: 10 **Line:** 193-195

Comment:6.

Please confirm whether the study has been approved by any local ethics committee.

Response:

Thank you so much for your comment. When I submitted the data collection request letter approved by the faculty of graduate studies (FGS), Mahidol University addressed it to the civil surgeon (head of health services at the district level). He wanted to check my ethical permission paperwork and rebate my local ethical approval after reviewing the documents, as I had Mahidol University ethical (IRB) approval.
