# Supplementary material for: The reality of embedded drug purchasing practices: Understanding the sociocultural and economic aspects of the use of medicines in Bangladesh
Source: PLoS One. 2023 Jun 28;18(6):e0287009. doi: 10.1371/journal.pone.0287009 (PMC10306190; doi:10.1371/journal.pone.0287009)
Supplement: Attachment — Submitted filename: Response to reviewer.docx [file pone.0287009.s002.docx]

Dear Academic Editor/Mary Hamer Hodges,
PLOS ONE journal

We sincerely thank you for the constructive comments and the opportunity to revise our manuscript ID: PONE-D-22-12723R1 entitled “**The Reality of Embedded Drug Purchasing Practices: Understanding the Sociocultural and Economic Aspects of the Use of Medicines in Bangladesh’**” for publication consideration in the journal of PLOS ONE. We made the revisions carefully and incorporate them into our revised manuscript in accordance with the comments. Our great pleasure is that the reviewer (s) have recommended our manuscript for publication with major revisions. Details of changes are listed as follows:

Journal additional requirements.

1. Please ensure that your manuscript meets PLOS ONE's style requirements, including those for file naming. The PLOS ONE style templates can be found at

**Response:** Checked accordingly. Thank you so much.

https://journals.plos.org/plosone/s/file?id=wjVg/PLOSOne_formatting_sample_main_body.pdf and

<https://journals.plos.org/plosone/s/file?id=ba62/PLOSOne_formatting_sample_title_authors_affiliations.pdf>

2. During our internal checks, the in-house editorial staff noted that you conducted research or obtained samples in another country. Please check the relevant national regulations and laws applying to foreign researchers and state whether you obtained the required permits and approvals. Please address this in your ethics statement in both the manuscript and submission information. In addition, please ensure that you have suitably acknowledged the contributions of any local collaborators involved in this work in your authorship list and/or Acknowledgements. Authorship criteria is based on the International Committee of Medical Journal Editors (ICMJE) Uniform Requirements for Manuscripts Submitted to Biomedical Journals - for further information please see here: <https://journals.plos.org/plosone/s/authorship>.

**Response:** Thank you so much for your note. For conducting this research, the researcher got a request letter for permission to collect research data in Bangladesh addressing the head of the health sector at the district level for conducting the research (see the request letter for permission to collect research data as attachment) from Dean, Faculty of Graduate Studies, Mahidol University, Thailand. The district's head of the health sector approved the request to collect research data as the study had already received the certificate of ethical approval (Protocol No. MU-CIRB 2020/237.3108) from the Mahidol University-Central Institutional Review Board.

3. Thank you for stating the following financial disclosure:

“Yes partial for conducting the fieldwork but the funder has no role in study design, data collection and analysis, decision to publish or preparation of the manuscript”

At this time, please address the following queries:

a)        Please clarify the sources of funding (financial or material support) for your study. List the grants or organizations that supported your study, including funding received from your institution.

b)        State what role the funders took in the study. If the funders had no role in your study, please state: “The funders had no role in study design, data collection and analysis, decision to publish, or preparation of the manuscript.”

c)        If any authors received a salary from any of your funders, please state which authors and which funders.

d)        If you did not receive any funding for this study, please state: “The authors received no specific funding for this work.”

Please include your amended statements within your cover letter; we will change the online submission form on your behalf.

**Response:** Thank you so much for the queries. Yes, included the statement in the cover letter:” The funders had no role in study design, data collection and analysis, decision to publish, or preparation of manuscript”.

4. Thank you for stating the following in your Competing Interests section:

“None”

Please complete your Competing Interests on the online submission form to state any Competing Interests. If you have no competing interests, please state "The authors have declared that no competing interests exist.", as detailed online in our guide for authors at http://journals.plos.org/plosone/s/submit-now

This information should be included in your cover letter; we will change the online submission form on your behalf.

**Response:** Yes, we included the state: "The authors have declared that no competing interests exist." in the cover letter.

5. We note that you have indicated that data from this study are available upon request. PLOS only allows data to be available upon request if there are legal or ethical restrictions on sharing data publicly. For more information on unacceptable data access restrictions, please see http://journals.plos.org/plosone/s/data-availability#loc-unacceptable-data-access-restrictions.

In your revised cover letter, please address the following prompts:

a) If there are ethical or legal restrictions on sharing a de-identified data set, please explain them in detail (e.g., data contain potentially sensitive information, data are owned by a third-party organization, etc.) and who has imposed them (e.g., an ethics committee). Please also provide contact information for a data access committee, ethics committee, or other institutional body to which data requests may be sent.

b) If there are no restrictions, please upload the minimal anonymized data set necessary to replicate your study findings as either Supporting Information files or to a stable, public repository and provide us with the relevant URLs, DOIs, or accession numbers. For a list of acceptable repositories, please see http://journals.plos.org/plosone/s/data-availability#loc-recommended-repositories.

We will update your Data Availability statement on your behalf to reflect the information you provide.

**Response:** Yes, there are ethical restrictions on sharing the data set according to the Mahidol University Central Institute of Review Board (MU-CIRB) as data contain potentially sensitive information. We revised this in our cover letter.

6. Please include captions for your Supporting Information files at the end of your manuscript, and update any in-text citations to match accordingly. Please see our Supporting Information guidelines for more information: <http://journals.plos.org/plosone/s/supporting-information>.

**Response:** Thank you so much for the suggestion. We revised according.

7. Please include a copy of Table 1 which you refer to in your text on page 8.

**Response:** We included the Table 1.

Additional Editor Comments:


Reviewers' comments:

Reviewer's Responses to Questions

**Comments to the Author**

1. Is the manuscript technically sound, and do the data support the conclusions?

The manuscript must describe a technically sound piece of scientific research with data that supports the conclusions. Experiments must have been conducted rigorously, with appropriate controls, replication, and sample sizes. The conclusions must be drawn appropriately based on the data presented.

Reviewer #1: Partly

2. Has the statistical analysis been performed appropriately and rigorously?

Reviewer #1: N/A

3. Have the authors made all data underlying the findings in their manuscript fully available?

The [PLOS Data policy](http://www.plosone.org/static/policies.action#sharing) requires authors to make all data underlying the findings described in their manuscript fully available without restriction, with rare exception (please refer to the Data Availability Statement in the manuscript PDF file). The data should be provided as part of the manuscript or its supporting information, or deposited to a public repository. For example, in addition to summary statistics, the data points behind means, medians and variance measures should be available. If there are restrictions on publicly sharing data—e.g. participant privacy or use of data from a third party—those must be specified.

Reviewer #1: No

4. Is the manuscript presented in an intelligible fashion and written in standard English?
PLOS ONE does not copyedit accepted manuscripts, so the language in submitted articles must be clear, correct, and unambiguous. Any typographical or grammatical errors should be corrected at revision, so please note any specific errors here.

Reviewer #1: Yes

5. Review Comments to the Author
Please use the space provided to explain your answers to the questions above. You may also include additional comments for the author, including concerns about dual publication, research ethics, or publication ethics. (Please upload your review as an attachment if it exceeds 20,000 characters)

6. PLOS authors have the option to publish the peer review history of their article ([what does this mean?](https://journals.plos.org/plosone/s/editorial-and-peer-review-process#loc-peer-review-history)). If published, this will include your full peer review and any attached files.
If you choose “no”, your identity will remain anonymous but your review may still be made public.

**Do you want your identity to be public for this peer review?** For information about this choice, including consent withdrawal, please see our [Privacy Policy](https://www.plos.org/privacy-policy).

Reviewer #1: No

**Response to reviewer comments**

Reviewer#1: Comments
This article explored an interesting and important aspects of drug purchasing practices in Bangladesh. However, one of the important flaws of this paper it tries to emphasis the vague quantitative findings through qualitative method without taking into account any quantitative factors. I would suggest the author to focus on the findings on the reasons why this practices rather than emphasizing on the 30 background factors e.g., Income, education, cultural beliefs, confidence in sellers, among others to make a conclusion.

**Response:** Thank you for your comment. In the revised version, we deleted the quantitative findings.

**Page no**.: 2, line no. 30-31.

Abstract
1. The author mentioned that “This study explores patterns of drug purchasing which underlie the socio-cultural and economic aspects of a Bangladeshi city”. How is it feasible to explore pattern through a qualitative study?

**Response:** Thank you so much for the comment and suggestions. We revised the abstract as “This study explores the drug purchasing practices in place of patterns of drug purchasing.

**Page no.**: 1, Line no. 16.

2. The results seemed to be vague quantitative findings without any specific numbers.

**Response:** Thank you so much for your comments. We revised and deleted the quantitative findings.

Background
1. Line 46: “….aggressive competition among the drug firms” this should be supported by evidence.

**Response:** Thank you so much for the comment. We have checked and included the reference as no. 1, namely Seeberg (2012). Apologies for the mistake. Noted that this inclusion of the new reference brought changes to the numbering of the entire manuscript references. Thanks for understanding.

**Page no**.: 3, Line no.: 46.

2. Line 52: “Moreover, every year, US$ 42 billion is spent on the inappropriate use of drugs” Where?

**Response:** We have checked the reference and corrected it as globally.

**Page no**. 3, Line no. 52.

3. Line 73: “For most patients in developing countries like Bangladesh…. what proportion? Any information?

**Response:** Thank you for the comment. We checked the reference and found that the article reported the proportion as the majority of the patient. We included ‘majority of’ and ‘outside of the home.’

**Page no**. 4, Line no. 74-75.

4. Line 88: ““access to safe, effective, quality and affordable medicines and vaccines for all”” I do not understand how the author has linked "access" with the context of current manuscript. Is this author meant the availability of quality drugs? If yes, is availability of quality drugs in pharmacy is similar to access? My understanding is that ‘access’ is determined by various socioeconomic factors and different from availability.

**Response:** Thank you so much for the comments. Here, we tried to interlink the term access is the people’s capacity to purchase medicine safely according to their wish because around 80% of people in Bangladesh have to buy drugs from the shortly trained drug seller.

5. Line 97: is not clear. What the author meant by density? Per facility?

**Response:** Thank you very much for the comment. Here, we used the ‘densely’ term from a reference that referred to the number of people (1045) living per square kilometer, not the per facility. We revised it accordingly.

**Page no**.: 5, Line no.: 96-97

6. Line 99: “Following field study techniques of organizing complex research subjects and data, three topics were chosen as collection tools, namely 'places, people, and events.” Should be supported by evidence of where this technique has been used?

**Response:** We have corrected it. We mistakenly separated the sentences, but it was one sentence and had the evidence at the end of the sentence.

**Page no**.: 5, Line no.: 102-103.

7. Line 186-187: “The opening hours of these drug stores were between 8.00 am and 10.am, however, they visited customers at home in an emergency.” This line is not clear. What the author wanted to mean here?

**Response:** Thank you very much for the comment. We reorganized the sentences. The first sentence is about the opening to closing hours. The second sentence was about the drug sellers visiting the client’s residence during the emergency call by the client.

**Page no**.: 10, Line no.: 191-192.

8. Line 181: the author mentioned that “three different drug stores were selected from three different settings?” It is not clear how the author determined different settings in terms of what? and why the author have chosen three different settings?

**Response:** Thank you so much for the comment. We have revised it. The settings of the drug shops were selected based on the location of the drug store, sellers training and clients flow.

**Page no**.: 10, Line no.: 183-187.

9. Line 195: author mentioned that “Customers’ drug purchasing behavior was influenced by demographic factors such as income, education, cultural beliefs and social network of the community people.” it is predictable that these socioeconomic factors influence customers’ drug purchasing behavior. However, what is new here? It would be useful to identify to what extent which factors affect the behaviour of the consumers? Otherwise, this statement remains vague, and dose not add anything to the findings. I assume this may not be possible to quantify this finding from only 30 interviews. I would recommend author not to focus on only the factors those affect the consumer drug purchasing behavior rather identify the key reasons for the identified behavior.

**Response:** Thank you so much for the comments. Yes, we all agreed with your suggestions and deleted the demographic factors. We revised following your comments.

**Page no**.: 10-11, Line no.: 199-201.

10. Line 196: “Those living in wealthier areas mostly bought prescription and trade name drugs” I do not see any calculation of wealth quintile. How the author concluded this is not clear. A clear representation of the calculation of wealth quintile should be presented. Or on what criterion the author divided the respondents into different socioeconomic group should be mentioned clearly.

**Response:** Thank you so much for the comments. We are extremely sorry for the term. The term wealthier replaced into the apartment building living areas as several drug sellers mentioned that.

**Page no**.: 11, Line no.: 201-202.

11. Line 207-208: please see comment 09.

**Response:** Thank you so much for the comment. We revised the sentences as ‘practices’ in place of patterns and ‘reasons’ in place of factors following the comment.

**Page no**.: 12, Line no.: 209, 212

12. Line 225: “customer’s individual choices” I am not sure how the author determined the choices of consumer? Did the author wanted to mean their previous behaviour of drug purchasing?

**Response:** Thank you so much. We have checked our data and revised the term ‘wishes’ in place of choice. Thanks again.

**Page no**.: 13, Line no.: 233.

13. Line 246: “In addition, the customer also seeks to buy a package of expensive drugs by instalment payments as well as by buying recommended drugs and those for a particular ailment.” This finding is interesting. However, the author ha not mentioned what is the understanding between the consumer and the drug seller regarding EMI? How the expensive drugs will be sold to consumers in EMI without any guarantee of repayment by the consumers?

**Response:** Thank you very for much for the comment. We revised and included about the understanding of medicine installment package according to our field data. Our data revealed that the client do not buy full package of medicine at once but rather buy on daily basis installment with cash payment.

**Page no**. 14: Line no.: 256-259.

14. Line 252: “around $3 @ 85 takas per $” The author should mention which dollar they referred to.

**Response:** We have added the USD in the revised version. **Page no**.: 14, Line no.: 263.

15. Line 278: “Findings revealed that some unregistered local drugs were dispensed and sold in the drug store.” I think it is difficult to conclude this finding from information from only three drug stores.

**Response:** Thank you very for the comment. We revised this following the field data that some of the drug sellers dispensed and sold some unregistered local medicines that are not listed (registered) in the drug administration.

**Page no**.:15, Line no.: 289-290.

16. Line 406: “patient preference” the preference is a technical term, which should be assessed as revealed or stated. I would rather suggest the author to use behavior instead of preference.

**Response:** We revised this as ‘behavior’ instead of preference following your suggestion. Thank you so much.

**Page no**.: 21, Line no.: 419.

We sincerely thank you for the constructive comments and the opportunity to revise our manuscript. We sincerely hope that our responses are acceptable to you and the revised manuscript meets the PLOS ONE standard. If you have any issues with our answers or the revised manuscript, please let us know if I can further serve you. We will follow our guidance accordingly. Again, please allow us to express our gratitude for allowing us to revise our manuscript for consideration for publication in PLOS ONE.

We uploaded the files namely cover letter, manuscript (clear version), Revised articles with changes highlighted, response to reviewers, and a request letter of permission for research data collection.

Best regards

Md. Shahgahan Miah, Ph.D.

Email: [shahgahan-anp@sust.edu](mailto:shahgahan-anp@sust.edu)

Department of Society and Health

Faculty of Social Sciences and Humanities

Mahidol University, Thailand
