# Supplementary material for: The reality of embedded drug purchasing practices: Understanding the sociocultural and economic aspects of the use of medicines in Bangladesh
Source: PLoS One. 2023 Jun 28;18(6):e0287009. doi: 10.1371/journal.pone.0287009 (PMC10306190; doi:10.1371/journal.pone.0287009)
Supplement: Attachment — Submitted filename: Response to reviewer.docx [file pone.0287009.s003.docx]

**Response to Academic Editor**

**Comment**

Journal Requirements:

Please review your reference list to ensure that it is complete and correct. If you have cited papers that have been retracted, please include the rationale for doing so in the manuscript text, or remove these references and replace them with relevant current references. Any changes to the reference list should be mentioned in the rebuttal letter that accompanies your revised manuscript. If you need to cite a retracted article, indicate the article’s retracted status in the References list and also include a citation and full reference for the retraction notice.

**Response**

Thank you so much for the comment and suggestions. In response to your observation, we conducted an additional check, and the reference has been updated. At this point, it is hopefully complete and correct according to the requirements of the journal. For your kind information, in our last revised manuscript, we included some new articles according to the reviewer's comments. That is why the reference number changed, and we apologize for not mentioning it in our last response and rebuttal letter. Once again, I would like to thank you for your comments and suggestions.

**Additional Editor Comments:**

Apologies for taking the time to review your paper. Unfortunately, there is a limited number of reviewers who have the expertise and interest to review this paper. We have reviewers who initially agreed but later did not complete the review on time. I reviewed your paper and have following comments:

This is an interesting and important study that investigated the drug purchasing practices which underlie the socio-cultural and economic aspects of a Bangladeshi city. However, there are a few areas in the manuscripts that can be further improved considering my additional comments. I have following minor observations, please consider them while revising it:

**Response**

Thank you so much for considering our manuscript for your list and for your appreciation. Yes, we tried to address all your comments and observations, which really improved our manuscript.

**Comment:** 1.

In the first sentence of the abstract, please replace the word ‘standard’ with ‘common’. Please note that purchasing drugs with or without a prescription from retail drug shops is NOT a standard practice in Bangladesh, but it may be a common practice.

**Response**

Thank you so much for your valuable suggestion. Yes, we replaced it following your suggestion.

**Page:** 1	**Line:** 14

**Comment:**2.

Please add a sentence under the results of the abstract with the number of IDIs, KIIs and observations you have conducted.

**Response:**

Thank you so much for the comment. We double checked and found that we included the number of IDIs and KIIs in the methods section of the abstract. Yes, we added a sentence about the observation hours that we conducted during our fieldwork. Thanks again for the comment.

**Page:** 1	**Line:** 22-23

**Comment:** 3.

In your first revision, you did not highlight the texts in which you have made changes. Please do so in your next revision.

**Response:**

Thank you so much for the comment, and my apologies for the mistake. We followed your comments in our revised manuscript.

**Comment:**
**Page:** 4. Line 77, please define what it means about ‘C’ grade pharmacists.

**Response:**

Thank you so much for the comment. Yes, we included a sentence that explained the types of pharmacists in Bangladesh.

**Page:** 4	**Line:** 80-83

**Comment:** 5.

Line 190, please check again the time of the opening hours, whether it is 8.00 AM to 10.00 PM?

**Response:**

Yes, we double-checked and revised the opening hours. All three drug stores we investigated had regular hours of operation ranging from 8 a.m. to 10 p.m. However, they frequently open early and close late depending on the emergency needs of their clients. Thank you so much for the comment.

**Page:** 10	**Line:** 193-195

**Comment:**6.

Please confirm whether the study has been approved by any local ethics committee.

**Response:**

Thank you so much for your comment. When I submitted the data collection request letter approved by the faculty of graduate studies (FGS), Mahidol University addressed it to the civil surgeon (head of health services at the district level). He wanted to check my ethical permission paperwork and rebate my local ethical approval after reviewing the documents, as I had Mahidol University ethical (IRB) approval.
