# Supplementary material for: The reality of embedded drug purchasing practices: Understanding the sociocultural and economic aspects of the use of medicines in Bangladesh
Source: PLoS One. 2023 Jun 28;18(6):e0287009. doi: 10.1371/journal.pone.0287009 (PMC10306190; doi:10.1371/journal.pone.0287009)
Supplement: Attachment — Submitted filename: Response to reviewers.docx [file pone.0287009.s005.docx]

Dear Editor,
Thank you very much for your comments. We have revised our manuscript according to your
suggestions. We hope this revision is acceptable for you.

We address the comments specifically below: Reviewers' comments:

Reviewer's Responses to Questions

Comments to the Author

1. If the authors have adequately addressed your comments raised in a previous round of review and you feel that this manuscript is now acceptable for publication, you may indicate that here to bypass the “Comments to the Author” section, enter your conflict of interest statement in the “Confidential to Editor” section, and submit your "Accept" recommendation.

Reviewer #1: All comments have been addressed

Reviewer #2: (No Response)

2. Is the manuscript technically sound, and do the data support the conclusions?

The manuscript must describe a technically sound piece of scientific research with data that supports the conclusions. Experiments must have been conducted rigorously, with appropriate controls, replication, and sample sizes. The conclusions must be drawn appropriately based on the data presented.

Reviewer #1: Partly

Reviewer #2: Partly

3. Has the statistical analysis been performed appropriately and rigorously?

Reviewer #1: I Don't Know

Reviewer #2: N/A

4. Have the authors made all data underlying the findings in their manuscript fully available?

The [PLOS Data policy](http://www.plosone.org/static/policies.action#sharing) requires authors to make all data underlying the findings described in their manuscript fully available without restriction, with rare exception (please refer to the Data Availability Statement in the manuscript PDF file). The data should be provided as part of the manuscript or its supporting information, or deposited to a public repository. For example, in addition to summary statistics, the data points behind means, medians and variance measures should be available. If there are restrictions on publicly sharing data—e.g. participant privacy or use of data from a third party—those must be specified.

Reviewer #1: Yes

Reviewer #2: No

5. Is the manuscript presented in an intelligible fashion and written in standard English?

PLOS ONE does not copyedit accepted manuscripts, so the language in submitted articles must be clear, correct, and unambiguous. Any typographical or grammatical errors should be corrected at revision, so please note any specific errors here.

Reviewer #1: Yes

Reviewer #2: No

6. Review Comments to the Author

Please use the space provided to explain your answers to the questions above. You may also include additional comments for the author, including concerns about dual publication, research ethics, or publication ethics. (Please upload your review as an attachment if it exceeds 20,000 characters)

Reviewer #2: 

**Reviewer Comments and response:**     

| **Reviewer 1** | | |
| --- | --- | --- |
| **No.** | **Comments** | **Page and line number in the revised manuscript** |
| **1.** | **Comment:**  Many thanks for addressing the earlier comments, I have some further comments and suggestions attached in the manuscript.  **Response:**  Thank you so much for your valuable feedback to our manuscript. We tried our best to address all the comments and suggestions you made. | **N/A** |
| **2.** | **Comment:**  PONE-D-22-12723R2 review  The manuscript outlines a qualitative research study that explores drug seller and drug purchaser perspectives in Bangladesh. As I have stated, below, in my comments to the authors, literature on the topic from Bangladesh has not be explored and therefore there is overlap with previous findings. The authors need to revise their literature review and re-frame the introduction. This will allow the authors to focus on new findings rather than be repetitive of previous qualitative studies conducted in Bangladesh. I have added some papers for consideration but this is in no way exhaustive, just the ones with which I have some familiarity.  There are several comments on improving the English expression and adding more detail to improve manuscript clarity.  Because there are new findings that are worth highlighting, I think that the manuscript may be appropriate for publication after a significant revision.  **Response:**  Thank you very much for your comments and suggestions. We truly agree that the manuscript needs significant revisions with clear point and focus. We carefully revised it more consistently and respond to all of the comments and suggestions that you made. We really hope the revised manuscript addresses your concern.  We additionally revised some issues, including the affiliation of the first author. As a corresponding author, we did this along with some grammatical edits. | **N/A** |
| **Abstract** | | |
| 1. | **Comment:**  Line 22: Are medical representatives pharmaceutical company employees? Please provide a more intuitive description to the international reader.  **Response:**  Thanks for your helpful suggestion. We revised this as pharmaceutical company representative in place of medical representative. | Page no. 1  Line no. 22 |
| 2 | **Comment:**  Line 23:  a. nonverbal behaviour is a vague term. Add some specifics.  b. The next sentence implies that a single participant was selected. Please revise this and indicate the total number.  **Response:**  We are really sorry for making you confused by the unclear sentence. The sentence is modified to make it more evident and to ensure clarity.   a. We observed the conversations and interactions for medicine purchases between the drug seller and buyer. b. In particular, ‘participant’ here was mistakenly remained singular, so, we now corrected it to ‘participants’ and included the total number ‘A total of 40’. Additionally, we revised the last word analyzed thematically.   We hope that you find this revision clear. | Page no. 1  Line no. 23  Page no. 1  Line no. 23-24, 25 |
| 3 | **Comment:**  Line 28: use the active voice. You are describing the results overall here. So, starting a sentence with 'results revealed' is not informative. In qualitative studies it is useful to include the type of data collection method that the results were drawn from. Do you mean 'We detected that xxxxxx from IDI thematic content analysis?'  **Response:**  Yes, we have revised the sentence which included the ‘we found through thematic analysis following your suggestions. Thank you so much for the comments and suggestions. | Page no. 2  Line no. 28 |
| 4 | **Comment:**  Line 29: this sentence contradicts the first. You mean to describe the range of intentions of the customers. Revise the English expression to reflect this.  **Response:**  Yes, the revised sentence included ‘among the 30 participants, most individuals come without…”. Thank you so much for the suggestions. | Page no. 2  Line no. 29 |
| 5 | **Comment:**  Line 31: it would be more useful to include at least one cultural belief. Using the term 'cultural belief' is vague and general.  **Response:**  We double checked our data and include the cultural beliefs of buying medicines in full or partial course of doses, with or without prescription following your suggestion.  For better clarity, we also revised the sentence ..experiences of medications shape the… | Page no. 2  Line no. 31-32    Line: 33 |
| 6 | **Comment:**  Line 35: It is impossible to determine which were common versus rare behaviours. A semi-quantitative approach would be more meaningful. Some, most, many (with number of respondents) is often used in qualitative studies to indicate common vs. rare behaviour.  **Response:**  Thank you so much for your comment. We feel that putting the number of responses will clearly state the reflection of data. In this section, we revised the sentences following your suggestions and field data. We included few customers (n=7) sought drugs… a. many of the clients (n = 13) bought drugs through installment payments and with loans. | Page no. 2  Line no. 34,36 |
|  | **Comment:**  Line 37: the conclusion does not follow from the findings included in the results section. Be specific about what the findings can tell policy makers. This conclusion could have been written without conducting the study.  **Response:**  Yes, we revised the conclusion in the abstract following your comments and suggestions. We tried to reorganize the conclusion in line with the study aim. | Page no. 2    Line no. 39-43 |
| Introduction | | |
|  | **Comment:**  Line 86: I disagree that there is little research on sociocultural and economic factors that contribute to economic aspects of drug purchasing. Quite a bit has been done on antibiotic purchasing alone, in Asia (including Bangladesh) and Africa, among others. A more detailed literature review is needed. This probably should have been done before developing IDI question guidelines.  **Response:**  Thank you for your comment. In the revised version, we deleted the word little research and revised the sentence following the suggestions.  We reviewed the literature you suggested, thanks for your suggestion. From this literature review, we have added relevant information to contextualize the research. | Page no. 5  Line no. 95  Page no. 4, 5  Line no. 71-73, 93-95 |
|  | **Comment:**  Line 90: change 'gather' to 'gathered',  **Response:**  Thank you so much for the comment and suggestion. We have corrected it. | Page no. 5  Line no. 99 |
| **Methods** | | |
|  | **Comment:**  Line 109: it is known that different types of customers visit at different times of the day. Please provide some information on timing of preliminary observations.  **Response:**  Thank you for your suggestion. We double checked our data and included drug purchasing timing information as the reviewer suggested. | Page no. 6  Line no. 122-125 |
|  | **Comment:**  Line 118: Since there were only three outlets, I am assuming that the authors mean 'one each of a seller with limited training, a RMP and a trainee sales assistant.  **Response:**  Thank you for your understanding and feedback. We have revised the sentence included one each of a seller with ‘C’ (3 months training) grade pharmacist, rural medical practitioner (RMP) and trainee sales assistant following the comment. | Page no. 6  Line no. 132- 136 |
|  | **Comment:**  Line 122: this information should be in the results, not methods.  **Response:**  Thank you so much for the comment and suggestion. We removed this from the methods following the suggestions as this information already in the result section. | Page no. 11  Line no. 228-231 |
|  | **Comment:**  Line 129: please provide the number of each participant type.  **Response:**  Thank you for your suggestions. Yes, we provided the number of each participant type. | Page no. 7  Line no. 143- 145 |
|  | **Comment:**  Line 130: by primary healthcare providers with drug sellers, do you mean doctors that have a room in the drug shop??  **Response:**  Thank you for the query. Apology for the mistake and have corrected primary healthcare providers with drug sellers as drug sellers. We also corrected lay patients as patient, very experienced sales assistant as experienced sales assistants in the text and table as well. | Page no. 7, 9  Line no. 143-145 |
|  | **Comment:**  Line 141: For an international audience, some context or photo of what comprises a drug shop in Bangladesh would be useful. For many in the Western world, they think this means a fully heated/air-conditioned 'pukka' premise with tiled floors etc.  **Response:**  Thank you so much for your feedback. We included four photos of a drug store.  We double-checked and included information about the context of drug stores in the revised version. | Page no. 7,  Line no. 152  Page no. 11 Line no. 209-211 |
|  | **Comment:**  Line 146: what questions examined economic situations of the participants? This is a finding that you have described. Was affordability a topic that came up without a prompt?  **Response:**  Yes, we revised the sentence following the comments as we had questions about cost (ID guideline) of drug purchasing experiences to understand the economic situations of the participants. Thank you for the comment and suggestion. | Page no. 7  Line no. 161 |
|  | **Comment:**  Line 148: what do you mean by local cultural drug purchasing?  **Response:**  Thank you so much for the query. We explained about the local cultural beliefs of medicine purchasing practices of buying full or partial course of drug dose with or without prescription. | Page no. 8  Line no. 163-164 |
|  | **Comment:**  Line 149: as indicated for the abstract, define what is meant by non-verbal interactions (note that this is in the manuscript both with and without a hyphen- ensure consistency throughout)  **Response:**  Apology for the mistakes and we deleted non-verbal term. Thank you so much for the comment and suggestions | Page no. 8  Line no. 165 |
|  | **Comment:**  Line 154: what is meant by 'member checking'.  Ethical issues: it is uncommon for a study conducted in Bangladesh to not receive any local ethical approval. That form another country is often considered inadequate.  **Response:**  Thank you so much for the query. We mean the member checking about participant validation to ensure the data consistency with participants.  In our past reviewer comments, we responded that we showed our ethical approval letter and the data collection request letter approved by the faculty of graduate studies (FGS), Mahidol University, addressed to the civil surgeon (head of health services at the district level, Bangladesh). The civil surgeon rebates our local ethical approval after reviewing the documents, as I had Mahidol University ethical (IRB) approval. | Page no. 8  Line no. 170-171  N/A |
| **Results** | | |
|  | **Comment:**  Table 1:   - Omit the first sentence. Start with the total number of participants then briefly describe their characteristics without repeating all of the data in the table. - some numbers are in bold and others not. I don't see a consistent pattern. - were there no data on where the key informants resided? - I think that a lay patient is just a patient. - age 45+/-9 seems to be in the wrong column for education, it would be better if the year categories lined up for IDI and KII   **Response:**   - Yes, we have deleted the first sentence and started with to total number of participants as you suggested. - We non-bolded all the number in the table. - We double checked and found the data about key informant residence where 8 were resided in suburb and 2 were in slum. - Yes, we revised the lay patient as only patient following your suggestion. - We revised the table following your suggestions. Thanks again for your comments and suggestions.   Thank you so much for the comment and suggestion. | Page no. 9  Line no. 189  Line no. 200 |
|  | **Comment:**  Line 195: approximate or typical closing hours would be useful to include.  **Response:**  We checked our data and revised the approximate closing hours was 11p.m. Thank you so much for the suggestion. | Page no. 11  Line no. 214 |
|  | **Comment:**  Line 203: It is difficult to determine whether behaviours described here and elsewhere in the manuscript were self-reported during IDIs or observed by researchers or both.  **Response:**  Thank you so much for the comments. Yes, we agreed and revised the sentence. We included the way customer purchase medicine is influenced by the cultural beliefs about medicine practices… | Page no. 11  Line no. 221 |
|  | **Comment:**   - Line 205: The new sentence starting here should be part of a new paragraph. This information is about socioeconomic differences.   Table 2:   - this is a description of the drug stores. Change the title to reflect this. Since there are only three drug stores (sometimes called retail drug shops, drug shops- be consistent throughout the manuscript), it should be 'slum area' rather than 'slum areas'. - It's not clear where the data in this table were derived from. Drug shop staff or customers or both? - change common health problem to commonly reported health problem. change 'selling products' to 'products sold'. Were these data from customers or drug shop staff or both.   **Response:**  Thank you so much for the comments and suggestions.   - Yes, we revise the paragraph following your suggestions. - Yes, we changed the title of the table 2 as demographics of retail drug stores: insights from drug shop staff and customers in accordance your comments. Apology for the inconsistencies. Yes, we revised as retail drug stores in throughout the manuscript. - Yes, we have corrected the word slum areas into slum area - Yes, we have corrected the word common health problem into commonly reported health problem, and selling products into products sold following the suggestions. We double checked with our data and included the retail drug shop staff and customers in the title of the table 2 as data derived from both drug shop staff and customers. | Page no. 12  Line no. 226-227  Line no. 233 |
|  | **Comment:**  Line 214 'Practices of' can be deleted.  **Response:**  Thank you for the comments. We revised the theme namely drug purchasing practices as different staff and customers involved in the process of buying and selling of medicine. | Page no. 14  Line no. 238 |
|  | **Comment:**  Line 216: this reads as if there is one individual. Fix the English. **Response:**  Apology for the mistake. We revised this as apartment building residents in place of the individual living in the apartment building. Thank you so much for the comment. | Page no. 13  Line no. 240 |
|  | **Comment:**  Line 218: Make the distinction between drugs for chronic conditions such as diabetes, hypertension etc and drugs for acute conditions e.g. infections. This is an important distinction.  **Response:**  Thank you so much for your important suggestions. We checked our data and really sorry that we have no data about disease based medicine purchases. We will consider this in our further study. Thanks again. | N/A |
|  | **Comment:**  Line 233: was this behaviour restricted to the slum dwellers or those visiting drug shop 2 only? It seems to be a common behaviour.  **Response:**  This behaviour reported among the low-income and illiterate customers in the drug shops 2 only. We included this in our revised version. | Page no. 14  Line no. 260 |
|  | **Comment:**  Line 250: include whether this was the case for all drug shops.  **Response:**  We double checked our data and found that the case was for the drug shop 2 only and slum people considered this. We revised this according. | Page no 14  Line no. 272 |
|  | **Comment:**  Line 283": this is not a good quotation. flucloxacillin is the name of the drug in fluclox'. **Response:**  Thank you so much for the comments. This quotation reflects the effort that the drug seller put into convincing the customers to dispense their preferred brand of medicine if they asked for medicine with a brand name.  Yes, the medicine was the same, but different products were from different drug firms. We have corrected the flucloxacillin to G-flucloxacillin (produced by Gonoshasthya Pharma) after checking the data. Flucox, produced by the ACI Pharma, In Bangladesh, medicine is prescribed, sold, and purchased based on the drug’s brand name instead of its generic name. Therefore, drug sellers often dispensed more profitable and low-profile brand drugs, saying they were the same medicine. As only the top 20 drug firms maintain good manufacturing practices (GMP) in Bangladesh, there might be chances of selling and buying substandard medicines. Thanks for the comment. | Page no. 16  Line no. 309-310 |
|  | **Comment:**  Line 293: do the authors mean the drug stores in the slum or the drug stores in the slum. Fix the English. **Response:**  Thank you so much for the comments and suggestions. We included drug stores in the slum and revised the writing errors, changed ‘sole’ into ‘sold’ and ‘from’ into ‘with’. | Page no. 16 Line no. 317- 318 |
|  | **Comment:**  Line 296: remove the word 'of'.  **Response:**  Yes, we deleted the word of following your suggestions. Thank you for the suggestion. | Page no. 16  Line no. 323 |
|  | **Comment:**  Line 300: this is an interesting finding that drug sellers feel more comfortable to recommend unregistered drugs to those from lower SES groups. It's worth including in the discussion.  **Response:**  Thank you so much for the comments. Yes, we included this in the discussion section following your comments on our revised manuscript. | Page no. 22  Line no. 453-455 |
|  | **Comment:**  Line 304: omit the phrase 'from the drug store’. Also omit 'Data revealed that the'  **Response:**  Yes, we omitted both the phrases following your suggestions | Page no. 17  Line no. 331 |
|  | **Comment:**  Line 308: do the authors mean that these data came from drug shop observations? I have not seen any reference to findings from this data collection exercise up to this point in the manuscript.  **Response:**  Thank you so much for the comments. Yes, these data came from the observations. For your kind information, we used other data from observations. | Page no. 15, 16, 19  Line no. 307, 286, 335, 371 |
|  | **Comment:**  line 320: circumcision is not just a procedure used by Muslims. It is also conducted by people from other religious groups and those without religious conviction. There's no need to describe this as a religious ritual.  **Response:**  We deleted muslim ritual following your suggestions. Thank you so much for the comments and suggestions. | Page no. 18  Line no. 347 |
|  | **Comment:**  Line 325: excellent quote.  **Response:**  Thank you so much for appreciation. | N/A |
|  | **Comment:**  Line 329: research data don't report, participants do. Fix this throughout. There are examples of ‘data showed’, results revealed etc. that need the expression revised.  **Response:**  Apology for the mistake. We revised this as our participants also reported. We tried to fix this throughout the manuscript. Thank you so much for the comments and suggestions. | Page no. 13, 15, 18, 19,  Line no. 242, 249, 259, 283, 356 |
|  | **Comment:**  Line 340: similarly use the active voice 'we found that customers often visited.......  **Response:**  Revised this following your suggestions. Thank you so much. | Page no. 18 Line no. 367 |
|  | **Comment:**  Line 344: in the methods, the authors describe that observations are for detecting non-verbal interactions.  **Response:**  Acknowledging our mistake about using the word non-verbal. We omitted these from the entire manuscript. Thank you so much for the comments. | N/A |
|  | **Comment:**  Line 346: there is a quotation mark missing. It should probably go after 'what should I do'.  **Response:**  Thank you so much for the comment. Yes, we included this in our revised manuscript. | Page no. 19  Line no. 373 |
|  | **Comment:**  Line 357: Competition to achieve a reputation for successful treatment: This business aspect of drug stores is well worth highlighting. Little attention is given to economic factors from the supply side. Often suggestions for antibiotic stewardship improvements, for example, assume that the supply side has bad motives. There’s nothing wrong in working to make a business thrive.  **Response:**  Yes, we agree with you that there is nothing wrong in working to make a business thrive but the drug seller explained antibiotic use may harm health. We checked with our data and included findings from the drug sellers that this competition might increase the over use of antibiotic. | Page no. 19  Line no. 390 |
|  | **Comment:**  I did not find results on non-verbal interactions. **Response:**  We deleted the term non-verbal from the revised manuscript. | N/A |
| **Discussion** | | |
|  | **Comment:**  The opening sentence is incorrect. See the following papers:  Revise the first paragraph accordingly. Also, from these papers, determine what is new and novel about the data presented in the current manuscript. The first paragraph states that ‘new aspects’ were detected, which suggests that the authors are familiar with other work on this topic. This may possibly be from other countries. Nevertheless, it somewhat contradicts the opening sentence.  **Response:**  We deleted the first sentence. We tried to follow the suggested references style for revising our discussion part.  We revised the first paragraph according to your comments and suggestions. | Page no. 20-21  Line no. 404-419 |
|  | **Comment:**  Line 379: this sentence is vague. Point to the results that support this sentence, which should probably be broken into two or more parts. **Response:**  We have deleted this sentence, we mainly focused on the practices of medicine sells and purchases. We tried to break down the sociocultural and economic issues of medicine practices in our revised manuscript. Thank you so much for the comment. | Page no. 20  Line no. 404-411 |
|  | **Comment:**  Line 387: this sentence is also not true, since there are published findings from Bangladesh and elsewhere that have reported this.  **Response:**  We deleted the words new aspects. We also revised this paragraph following the comments. We found compared our findings with suggested references. | Page no. 21  Line no. 416-424 |
|  | **Comment:**  Line 399: I think this is a citation which is a superscript rather than in parentheses. **Response:**  Apology for the mistake. We revised this. For your kind information, the list of references reordered as we included new references following your suggestions. | Page no. 21  Line no. 429 |
|  | **Comment:**  Line 403: this is new data that was not included in the results section. Move to results.  **Response:**  Thank you so much for the comment. Yes, this data already included in the results section. | Page no. 18  Line no. 356-363 |
|  | **Comment:**  Line 434: purposive sampling is not a limitation but a standard qualitative technique.  **Response:**  Yes, we agreed with you and apologize for the mistake. We deleted this and revised this limitation of the study section. | Page no. 23  Line no. 467-469 |
|  | **Comment:**  Conclusion: see the comment for the conclusion section in the abstract.  **Response:**  Thank you so much for the comments and suggestions. We revised the conclusion section following the comments. Thanks again. | Page no. 23-24  Line no. 476-489 |
|  | **Additional response**  For your kind information, we edited few issues in the discussion section-   - We have edited some grammatical issues in the discussion section. | |
|  | **Reviewer 2** |  |
|  | **Abstract** |  |
|  | **Comment:**  Line 39 ‘drug resistance’ not sure what the author meant here  **Response:**  Thank you for the comment. We revised this as reduce the effectiveness of medication instead of drug resistance for more clarity. | Page no. 2  Line no. 40-41 |
|  | **Comment:** Line 66 This line is not clear. Did the author want to mean "used to visit”?  **Response:**  Thank you so much for the comment. Yes, we try to mean ‘used to visit’ and revised accordingly. | Page no. 3  Line no. 69 |
|  | **Comment:** Line 67 patients where?  **Response:**  It was, Patients in Bangladesh. Revised accordingly | Page no. 4  Line no. 73-74 |
|  | **Comment:** Line 76 Can the author add any reference?  **Response:**  Thank you so much for the comments. We revised the number of 1,06,919 and cited from the reference [20]. Apology for the mistake. Thanks for understanding. | Page no. 4  Line no. 83 |
|  | **Comment:**  Line 87 This is the first time, author introduced two new terms. It would be great if author can define what actually meant in this paper by sociocultural and economic aspects. Is there any evidence in research that these two factors influence drug purchasing behavior? Can author include any conceptual framework or refer that how these factors may influence individual behavior while purchasing drugs? What is the hypothesis and how it is grounded on theory?  **Response:**  Thank you so much for your comments and suggestions. We used these two new terms in order to understand the medicine purchasing practices and how these are connected to sociocultural and economic aspects. What are the realities of expectation, negotiation, and decision-making, and how are medicines being dispensed and acquired between drug sellers and customers? The sociocultural and economic aspects of the hypothesis provide answers to these queries.  Sorry about the word of using factor. We included the word aspects instead of factor. As of our best knowledge, we didn’t find any evidence about these two factors influence drug purchasing behavior.  Yes, we included a conceptual framework that reflect the drug dispense and purchasing practices interlinked with the sociocultural and economic aspects.  Sorry, we did not used any theory in this research.  Thank again for the comments and suggestions. This really helps to make it more evident. | Page no. 5  Line no. 95-96    Page no. 11  Line no. 224 |
|  | **Comment:**  In line 87 What are the evidences that irrational drug buying exists?  **Response:**  The past research citation no. [16] identified the unnecessary buying and selling of drugs. We replaced the word unnecessary in place of irrational. | Page no. 5  Line no. 96 |
|  | **Comment:**  Line 95 The author did not mention anything about sociocultural and economic aspects rather than general description of qualitative analysis in the methodology. It may be mentioned how the author assessed these two aspects which are the prime focus of this manuscript?  **Response:**  Thank you so much for the comment. We tried to mention briefly about the sociocultural and economic aspects in the methods section. As we adopted the ethnographic method, we tried to explore the describe in details practices about drug purchasing and sells. For your information, we did not assessed quantitative indicators except some demographic information of the participants. We analyzed the practices of medicine as behavioral part. Thanks again. | Page no. 5  Line no. 115-118 |
|  | **Comment:**  Line 99 The author may clarify if there were any criterion for selecting study site?  **Response:**  We already clarify about the study area that is densely populated and poorest healthcare indicator in Bangladesh. Thank you for the comment. | Page no. 5  Line no. 108-109 |
|  | **Comment:**  Line 117 This line is confusing.  **Response:**  Apology for the mistake. We revised the sentence as participants were purposefully selected from three different retail drug stores where they worked as private merchants, primary healthcare professionals, or consumers. Thanks for the comment. | Page no. 6  Line no. 132-134 |
|  | **Comment:**  Line 128-132 Categories of the respondents seemed do not match with the reported table.  **Response:**  Yes, we revised table 1 accordingly. | N/A |
|  | **Comment:**  Table 1, What is the difference between clients and lay patients? Authors may consider two sides, supply (users) and demand (providers). Other classification is confusing.  **Response:**  Thank you so much for comment and suggestion. We included this as clients and patients. Clients who bought medicine for his own or others and the patient who present symptoms and bought medicine. We did not categorize supply and demand sides. Thanks again for the suggestion, will consider for further study. | Page no. 9  Line no. 200 |
|  | **Comment:**  Line 185, This section seemed to be a mix of background, methods, and results in the result section, the author may reorganize it.  **Response:**  Thank you so much for the comment. Yes, we agree with you. We tried to explain about the context through this mixed description. Thanks for the comment. | N/A |
|  | **Comment:**  Line 203 t is unclear how the authors concluded these findings? Where is the data? How many of the respondents mentioned these aspects? How the authors measured their cultural beliefs?  **Response:**  Apology for the mistake. We deleted the customers drug purchasing behavior. We revised this as the way customer purchase medicine is influenced by…..  We did not measure their beliefs rather describe their cultural practices of buying medicine. | Page no. 11  Line no. 221 |
|  | **Comment:**  Table 2 t may be but would be strong to say that a drug store being in an affluent area, their clients will be from higher income group. Did the authors analyzed the characteristics of the respondents who visited these drug stores? There might be several factors for a client to visit a drug store, one of them is drugs availability. Poor people may also visit to drug seller in the affluent area as they might sell drugs which are not available in other place or maybe there are other factors, easy to reach, give discounts.  **Response:**  Thank you so much for the comments and suggestions. As we did not measure the asset quantile, we just used the term higher income residential area considering the location of the drug store. We double-checked our data and found that the slum people used to visit their nearest drug store. | N/A |
|  | **Comment:**  Line 216 How did author obtained this information?  **Response:**  We obtained this information from the demographic data in particular the residential status during interview | Page no. 13  Line no. 240 |
|  | **Comment:**  Line 268 What if the drug is an antibiotic?  **Response:**  Thank you so much for the comment. Similar experiences shared in case of antibiotic. We included the quotation in case of antibiotic. | Page no. 15  Line no. 288-292 |
|  | **Discussion** |  |
|  | **Comment:**  Line 379 This is not well justified in this study how these linked with economic aspects. This should be clear in the method and grounded on any theory.  **Response:**  Yes, we deleted this and revised accordingly. Thank you so much for the comment. | Page no. 20  Line no. 404-408 |
|  | **Comment:**  Line 387, I am not sure if this is really a new aspect or regular practice of people with from low socioeconomic group.  **Response:**  We revised this sentence and reorganized accordingly. Thank you so much for the comment and suggestion. | Page no. 21  Line no. 415-419 |
|  | **Comment:**  In line 415 The author may also discuss the about the drug policy of Bangladesh and specifically where this study can contribute.  **Response:**  Thank you so much for the comment. We just briefly include the monitoring and regulation of the over the counter drugs following the national drug policy. | Page no. 22  Line no. 446-447 |
|  | **Comment:**  In line 427 This is not clear how the patients will be aware from this findings? Should policymakers take initiatives to aware the patients?  **Response:**  Yes, the policymakers could take the initiative of multilayer intervention to aware the patients. We found that drug sellers could play the vital role as health educator for the patients. | Page no. 21, 23  Line no. 434, 462-463 |
|  | **Comment:**  In line 437 The author may mention theoretical aspects related to healthcare belief and how it is influenced such as using health belief model or revise related terminologies.  **Response:**  We deleted the sentence and revised following the reviewers’ comments and suggestions. We did not adopt any theory in this study. Thank you so much for the comment. | Page no. 23  Line no. 467-469 |
|  | **Comment:**  In line 461 This seemed not clear, what author exactly want to mean or suggest?  **Response:**  Thank you so much for the comment. We tried to make it clear including the sentence as drug sells and purchases practices varied with urban settings of slum and suburb areas. Therefore, considering the local context of drug practices may implicate into the policy for ensuring safe and rational use of medicine. | Page no. 24    Line no. 493 |

7. PLOS authors have the option to publish the peer review history of their article ([what does this mean?](https://journals.plos.org/plosone/s/editorial-and-peer-review-process#loc-peer-review-history)). If published, this will include your full peer review and any attached files.


If you choose “no”, your identity will remain anonymous but your review may still be made public.


Do you want your identity to be public for this peer review? For information about this choice, including consent withdrawal, please see our [Privacy Policy](https://www.plos.org/privacy-policy).

Reviewer #1: No

Reviewer #2: No

Again, thank you very much for all your meaningful comments and suggestions. We hope that all of our responses address your concerns.

Sincerely yours,

Dr. Md. Shahgahan Miah

Associate Professor

Department of Anthropology

Shahjalal Univeristy of Science and Technology

Sylhet-3114, Bangladesh

Email: shahgahan-anp@sust.edu
